# Adolescents’ perspectives on non-pharmacological pain interventions for sickle cell crisis management: A population-based survey

**DOI:** 10.1371/journal.pone.0330127

**Published:** 2025-08-19

**Authors:** Bukola Mary Ibitoye, Bernie Garrett, Manon Ranger, Jennifer N. Stinson

**Affiliations:** 1 School of Nursing, University of British Columbia, Vancouver, British Columbia, Canada; 2 Child Health Evaluative Sciences, The Hospital for Sick Children, Toronto, Ontario, Canada; 3 Lawrence S. Bloomberg Faculty of Nursing, University of Toronto, Toronto, Ontario, Canada; Fred Hutch Cancer Center: Fred Hutchinson Cancer Center, UNITED STATES OF AMERICA

## Abstract

**Background & Aims:**

Nigeria has the highest sickle cell disease (SCD) prevalence globally. Research indicates that adolescents often utilize non-pharmacological interventions (NPIs) to cope with recurrent painful sickle cell crises (SCC) while navigating the complex biopsychosocial challenges of adolescence. This study explored the use and perceived value of NPIs for managing SCC pain from the perspectives of Nigerian adolescents, including their preferred media for NPI educational resources.

**Methods:**

A population-based survey targeted adolescents aged 12–18 living with SCD in Nigeria. Adolescents were recruited using convenience and snowballing sampling through SCD support groups and were asked to complete a 27-item questionnaire delivered online or on paper. Data were descriptively analysed.

**Results:**

Out of 138 surveys returned, 123 surveys were included in the final analysis. Among the participants, 51% were males with a mean age of 14.85 (SD ± 2.11). Most participants (77%) had used at least one NPI to manage pain during SCC, and 31 different NPIs were reported. The most used NPIs were massage (53%), herbal products (37%) and prayer (30%). Participants described various factors that impacted their use of NPI, including healthcare providers’ disapproval. The most common NPIs that the adolescents plan to use in the future were: herbal products (45%), massage (33%) and prayer (30%). Lastly, respondents preferred to receive NPI education via videos (34%) and animations (20%).

**Conclusions:**

The study showed that Nigerian adolescents use various NPIs to manage their pain during SCC, including traditional remedies and physical and spiritual interventions. Most participants already used (or planned to use) herbal products and other NPIs with limited scientific evidence of their safety or effectiveness on SCD outcomes. This warrants the attention of clinicians and researchers as there is an urgent need to further explore the specific NPIs used and their effectiveness on SCD outcomes and safety.

## Introduction

Sickle cell disease (SCD) is the most common inherited hemoglobinopathy worldwide. Approximately 515,000 new births annually, with 7·74 million people living with the disease globally [1]. The largest burden of the disease is in sub-Saharan Africa, where there were 405,000 births in 2021 and an all-age prevalence of 5·68 million cases, representing 73% of the global burden [1]. SCD represents a significant health problem in sub-Saharan Africa, particularly in Nigeria, where an estimated 150,000 babies are born with SCD in Nigeria each year, and it is the sixth cause of childhood death [2–5]. Nigeria is a low-middle-income country (LMIC) with limited health resources, which are disproportionally distributed across the large and ethnically diverse country. With high mortality rates due to infectious diseases, healthcare resources in Nigeria are often directed towards diseases such as malaria, HIV/AIDS and cholera, with limited focus on inherited chronic illnesses like SCD. Unsurprisingly, progress in SCD management has remained slow in Nigeria compared to high-income countries such as the U.S. and Belgium [6–8]. This slow progress can be attributed to the high prevalence of tropical infections such as malaria and the lack of (a) a national newborn screening program for SCD, (b) funding for SCD research, and (c) evidence-based interventions (such as hydroxyurea) to improve survival and SCD management [4,7,9].

The hallmark of SCD is sickle cell crisis (SCC), also known as vaso-occlusive crisis [10]. SCCs are recurrent, unpredictable and self-limiting episodes of moderate-severe acute pain in SCD [11,12]. The frequency of SCC peaks during adolescence, and it is the leading cause of hospitalization in young people with SCD [13]. Ongoing SCC pain is a complex and multifactorial phenomenon, rendering it an extremely difficult pain to treat [14]. Opioid analgesics are the first-line treatment of SCC pain [14]. These medications can have severe side effects, such as respiratory depression and drug dependence. Also, despite the potency of these drugs, SCD patients continue to express that their pain is often misunderstood and undertreated, and they are often stigmatized due to their high demands for analgesia [13,15,16]. In LMICs, there is limited access to opioids due to physicians’ unwillingness to prescribe, and the relatively high cost of potent analgesia contributes to sub-optimal pain management [17–19]. Experts strongly recommended that SCC pain be managed with pharmacological and non-pharmacological interventions (NPIs) to improve the pain outcomes of SCD patients during crises [20,21]. In this study, NPIs are defined as any intervention that can be used to manage SCC pain, apart from pharmacological agents and other conventional treatments such as surgery.

Researchers have reported that patients use various NPIs, with or without medications, to manage SCC pain. A recent scoping review identified 27 different NPIs used by pediatric SCD patients, with the most common ones used at home being prayer, massage, and distraction [22]. Notably, 85% of studies (n = 27) in the scoping review were conducted in the United States [22]. Only one study conducted in Nigeria reported that prayer and fluid intake were the NPIs used by children to manage their SCC [23]. Other researchers in Nigeria have explored the use of NPIs for the management of SCD in general without focusing on SCC pain [24,25]. Thus, it remains unclear what NPIs are employed by adolescents in Nigeria and other LMICs to help manage their crisis-related pain. Therefore, this study aimed to examine the use, prevalence, types, and perceived value of NPIs used to manage SCC pain in Nigerian adolescents, as well as their preferred medium for delivering NPI educational resources.

## Materials and methods

### Design

A population-based survey was conducted targeting adolescents living with SCD in Nigeria.

### Participants

Adolescents aged 12–18 years old living with SCD were targeted and recruited using convenience and snowballing sampling through SCD support groups such as Sickle Cell Celebs (Meta/Facebook). The inclusion criteria were adolescents aged 12–18 years, having any SCD genotype, having at least one episode of SCC in the past year, and being able to speak/read English. The exclusion criterion was adolescents with cognitive limitations or communication deficits that hindered participation. This study represents an inductive exploratory approach; hence, the sample size was estimated based on similar work [26]. A minimum of 120 adolescents was deemed appropriate based on a previous study [23].

### Data collection instrument

A 27-item questionnaire was divided into two main sections. The first section contained 13 questions which gathered information on the participants’ socio-demographic data, such as their age, sex, level of education, ethnicity, and health information (e.g., nature and severity of SCD, SCC, and SCC pain). This section included close-ended and open-ended questions and was developed based on similar studies exploring SCC pain in pediatric sickle cell patients [27,28]. The second section consisted of a Non-Pharmacological Pain Management Strategies Questionnaire, which was developed based on the findings of a prior scoping review on the use of NPIs in SCC pain in pediatrics [22] and guided by the “Which Health Approaches and Treatments Are you using?” (WHAT) questionnaire [29]. This questionnaire contained 12 items, entailing a mix of multi-choice, Likert scale, and open-ended questions (see S1 File). The survey also included the option to indicate interest in participating in a subsequent study as a part of this project.

The questionnaire was piloted with four Nigerian adolescents to assess clarity and suitability for the study’s purpose [30]. The readability was determined with the Flesch Reading Ease, a Flesch-Kincaid readability test, as it is commonly used in surveys in Nigeria [31,32]. The readability score of the questionnaire was 84.7%, indicating it can easily be read by an average 12-year-old [33]. Face validity was also determined through a review by experts in the area. Also, the questionnaire was reviewed by a Junior Secondary School teacher in Ilorin, Kwara State, and a Lecturer at the Department of English, University of Jos, to ensure clarity for Nigerian adolescents. The survey was revised based on the findings of this pre-testing, face validation and expert review process. Some changes included replacing certain terms with simpler terminology (e.g., “intervention” was replaced with “method”). Those who participated in the pre-testing were not included in the study sample.

### Data collection procedure

The questionnaires were distributed in two formats: online through Qualtrics (Qualtrics 2023, Provo, UT,USA) and in printed form. For the online format, the survey invitation and hyperlink were disseminated through posters and social media posts on sickle cell support groups nationwide and on Facebook/Meta, such as Sickle Cell Celebs and Nigeria Sickle Cell Warriors. As an incentive to participate, participants could enter to win one of ten 7000-naira cash prizes. A short set of screening questions were used, and only those eligible were redirected to the survey.

The printed surveys were distributed across eight nationwide SCD foundations and clinics, specifically in Kaduna, Kwara, Jigawa, Enugu, Kaduna, and Ogun States. Certain officials in these foundations and clinics were trained on the purpose of the study, the recruitment of participants, inclusion/exclusion criteria and survey administration. During their routine club and clinic days, the personnel advertised the survey to the adolescents. Interested participants were screened using a brief eligibility survey, and those eligible were given information about participation, the 7000-naira prize incentive, and the printed survey.

The survey (paper-based and online) was distributed between March 15^th^, 2023 – February 20^th^, 2024.

### Ethical review

The study was reviewed and approved by the University of British Columbia Behavioural Ethics Board (Canada; H23-00413) and the University of Ilorin Ethics Review Committee (Nigeria; ERC PAN/2023/02/0356). The questionnaires were anonymous, as no personal identifying information was collected in the survey (contact information for participation in the draw was not linked to the questionnaire, maintaining their anonymity). Consent information was provided at the beginning of the survey, and it was stated that submission of the survey was taken as their consent to participate in this research. The two ethics committees approved a parental consent waiver for the adolescents as the survey posed minimal risks to the participants and was anonymous.

### Inclusivity in global research

Additional information regarding the ethical, cultural, and scientific considerations specific to inclusivity in global research is included in the S2 File.

### Data analysis

Demographic and health information data (e.g., age, sex, and frequency of SCC) and close-ended responses to the Non-Pharmacological Pain Management Strategies Questionnaire were analyzed using descriptive statistics. Frequencies and percentages were calculated for discrete variables, and means and standard deviations were calculated for continuous variables. The Chi-square test was used to explore any associations between categorical variables. Consistent with prior recommendations, only NPIs with frequencies of five or more were included in the chi-square analysis (S3 File) [34,35]. The significance level was set to 0.05. The quantitative data from the survey were exported into SPSS Statistics (Version 27, IBM, NY, USA) for data analysis. The NPIs were categorised into three main groupings: biomedical, alternative health or combined interventions. Biomedical NPIs refer to interventions developed through biomedical science (based on the principles of biology, physics, and biochemistry) [36]. Reported biomedical NPIs were further logically classified into temperature modulation, physical manipulation & movement, neurologically stimulating activities, and rest. The Alternative health interventions category was used for interventions rooted in traditions and theories distinct from modern biomedical science (that claim mechanisms of action outside of those currently accepted by scientific and biomedical consensus) [36]. Alternative health interventions were further classified as spiritual, herbal, nutritional, and mind-body interventions. Combined interventions represent those NPIs combining both biomedical and alternative health NPIs, such as Yoga, having both exercise and spiritual elements.

The responses to the 17 open-ended questions, including close-ended questions with the “if others, please specify” response option, were analyzed using simple content analysis using Nvivo (Nvivo 12, Lumivero, CO, USA) qualitative data analysis tool. The narrative responses were read and re-read, and specific thematic elements were identified and allocated an appropriate code by the primary investigator. A coding list was continuously generated and edited until no new themes emerged from the data. Then, the codes were condensed into thematic categories and sub-categories. Other researchers (co-authors) further reviewed the narrative responses, coding, categories and sub-categories to confirm validity. The coded themes and NPIs identified were also ranked to identify and explore significant and common thematic elements related to NPI use.

## Results

**Fig 1:**
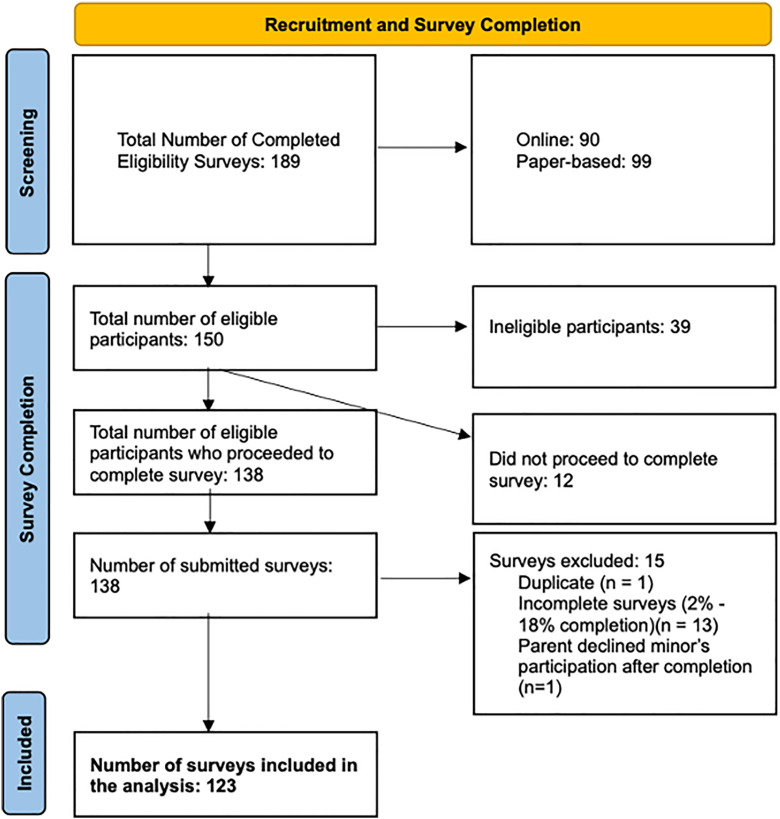
Flowchart for Recruitment and Survey Completion.

Initially, the only medium for collecting data was online surveys. After three months of disseminating the online survey, 24 surveys had been submitted, indicating a lower response rate than the anticipated 120 responses. Feedback from sickle cell foundations and clubs suggested that many adolescents had Internet access challenges. Hence, the printed format was subsequently implemented, and a total of 138 surveys were returned, and 123 surveys were returned for analysis (Fig 1).

### Quantitative analysis

#### Demographic data.

**Fig 2:**
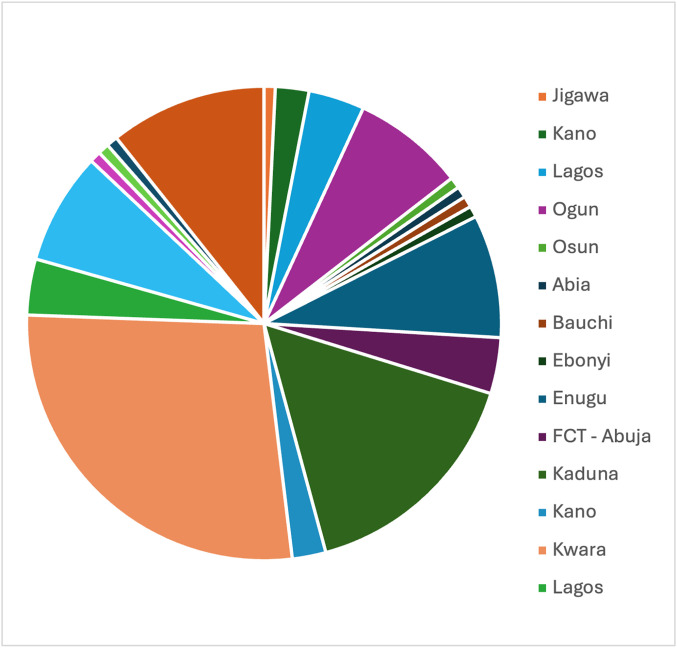
Respondents’ state of residence.

The participants were almost equally distributed between females (48.8%, n = 60) and males (51.2%, n = 63) with a mean age of 14.85 (SD ± 2.15, n = 123). They resided in 19 states and one territory (out of 37) in Nigeria, with the majority living in Kwara, Kaduna and Taraba (Fig 2). Participants were primarily secondary/high school students (74.0%, n = 91), Muslims (58.5%, n = 72) and had the homozygous allele, HbSS (89.4%, n = 110). They (n = 116) reported a total of 526 sickle cell crises (SCC) in the past year, with a mean of 4.53 (SD ± 3.01). Most incidents of SCCs (64%, n = 336) were managed in hospitals and 34% (n = 176) were managed at home. Participants recalled mean pain intensity rated on a Numerical Rating Scale during their last sickle cell crisis was 6.6 (SD ± 1.79) out of 10. The demographic and health information data of the participants are shown in Table 1.

**Table 1:** Demographic Characteristics (n = 123).

Characteristics	Frequency (%)	Mean (SD)
Age		14.85 (2.15)
Sex		
*Female*	60 (48.8)	
*Male*	63 (51.2)	
Education		
*None*	1 (0.8)	
*Primary*	11 (8.9)	
*Secondary*	91 (74)	
*University/Polytechnic/College of education*	20 (16.3)	
Occupation		
*Student*	119 (96.7)	
*Others*	3 (2.4)	
*Missing*	1 (0.8)	
Religion		
*Christianity*	47 (38.2)	
*Islam*	72 (58.5)	
*Prefer not to say*	1 (0.8)	
*Missing*	3 (2.4)	
Age of initial diagnosis		
*Birth*	10 (8.1)	
*0 - 5 years*	55 (44.7)	
*6 - 10 years*	42 (34.1)	
*11 - 15 years*	13 (10.6)	
*16 + years*	2 (1.6)	
*Missing*	1 (0.8)	
Genotype		
*Sickle cell anaemia (SS)*	110 (89.4)	
*Sickle haemoglobin C (SC)*	6 (4.9)	
*I don’t know*	7 (5.7)	
Total number of crises in the past year	526	4.53 (3.01)
Total number of crises managed in hospitals	336 (64)	3.14 (2.74)
Total number of crises managed at home	176 (34)	2.35 (2.4)
Pain intensity of last sickle cell crisis on NRS		6.6 (1.79)

NRS, numerical rating scale.

### Past use of NPIs

Most participants (76%, n = 94) had used at least one NPI to manage pain during SCC in the last year (Table 2). The major reasons for using NPI (n = 92) were “to feel better” (49%, n = 45) and “to prevent the pain from getting worse” (37%, n = 34). Aside from the prespecified options, other responses (9%, n = 8) include NPI serving as first aid and “to increase blood.” Of the 28 participants who had never used an NPI before, 24 provided reasons for not using them. The top reason for non-usage was “the healthcare team did not recommend it” (38%, n = 9). Two participants (8%) indicated other reasons, such as caregiver disapproval. Table 2 illustrates the reasons given for using/not using NPIs. 56% of the participants used 1–3 different types of NPI (one 35% n = 43; two 11% n = 13; three 8% n = 10), while 22% (n = 27) had used between 4–14 types of NPIs. The most used NPIs were massage (53%), herbal products (37%), prayer (30%), and heat application (20%). Nine participants indicated 13 additional NPIs they use to manage their sickle cell crisis, aside from the pre-defined list in the survey. Table 3 shows all NPIs identified by participants and their frequencies.

**Table 2:** Use of Non-Pharmacological Interventions (n = 123).

Variable	Frequency (%)
Past use of NPI	
*No*	28 (23)
*Yes*	94 (77)
Did you change your treatment due to NPI use?*	
*No*	76 (81)
*Yes*	15 (16)
*Missing*	3(3)
Do you have any difficulty using NPI?*	
*No*	73 (78)
*Yes*	14 (15)
*Missing*	7 (7)
Future use of NPI	
*No*	16 (13)
*Not sure*	37 (30)
*Yes*	64 (52)
*Missing*	6 (5)
Reasons for using NPIs	
*To feel better*	45 (49)
*To cure sickle cell crisis*	32 (35)
*To prevent the pain from getting worse*	34 (37)
*It is natural and safe*	20 (22)
*It was prescribed*	14 (12)
*Nothing else worked*	4 (4)
*Others*	8 (9)
Reasons for not using NPIs	
*The healthcare team did not recommend it*	9 (38)
*I believed it would not relieve my pain*	5 (21)
*I was afraid of the side effects or mixing it with my medical treatment*	6 (25)
*I did not want to use it*	3 (13)
*I did not have enough information about it*	3 (13)
*Others*	2 (8)

*Only those who answered Yes to using NPI in the past were asked to answer these questions.

**Table 3:** Types of Non-Pharmacological Interventions (n = 102).

NPI Category/Sub-Category	NPIs	Description	PAST USE OF NPIs	FUTURE USE OF NPIs
			Frequency (%)	Frequency (%)
**Biomedical**	***Temperature Modulation***			
	Local Heat	Applying heat to the painful area with hot compress/hot water bottle)	22 (24)*	16 (16)
	Warm bath	Having a bath/shower with warm water	19 (20)	16 (16) ^#^
	Infrared radiation	Application of infrared heat therapy to the painful area	2 (2)	–
	***Physical Manipulation & Movement***			
	Massage	Simple massage of painful areas by a therapist or self-administered	49 (53)*	34 (33)^#^
	Deep breathing exercises	Deep breathing exercises	11 (12)	4 (4)
	Progressive muscle relaxation	Progressive muscle relaxation exercises	12 (13)	10 (10)
	Physical Exercises	Exercise activity, e.g., walking around	3 (3)	–
	Massage with Robb	Massage with Robb (Eucalyptus, Camphor, Menthol, Salicylate and Pine oil)	1 (1.2)	–
	Massage with Shea butter	Massage with Shea Butter	1 (1.2)	–
	Massage with Palm kernel oil	Massage with Palm Oil	1 (1.2)	–
	Massage with Olive oil	Massage with Olive Oil	1 (1.2)	–
	Local pressure	Applying physical pressure to the painful area	1 (1.2)	–
	Physiotherapy	Physiotherapy with a clinician	1 (1.2)	–
	***Neurologically Stimulating Activities***			
	Distraction (videos and games)	Cognitive distraction (unspecified)	18 (19)	9 (4)
	Reading	Reading a book, magazine or comic book	8 (9)	10 (10)
	Music	Listening or playing music (unspecified)	5 (5)	5 (5)
	Animal activity	Playing with a pet	3 (3)	3 (3)
	Recreational activities	Unspecified recreational activity	3 (3)	4 (4)
	Virtual reality (VR)	Playing a VR game/experience	1 (1)	2 (2)
	Relaxation delivered through VR	Using virtual reality to achieve relaxation	–	2 (2)
	Watching football	Watching a football game	–	1 (1)
	***Rest***			
	Sleep/rest	Sleeping/Resting in a comfortable bed	20 (22)*	21 (21) ^#^
	Relaxation	Relaxing (unspecified)	8 (9)	6(6)
**Alternative Health**	***Spiritual interventions***			
	Prayer	Praying (by the SCD patient or relative)	28 (30) *	30 (30) ^#^
	***Herbal and nutritional interventions***			
	Herbal products	Use of unspecified herbal compounds and substances such as Akpuru Leaf (Ficus capensis) and ginger juice	36 (38) *	46 (45) ^#^
	Tomato paste with coke	Drinking Tomato Paste and Coca-Cola remedy	1 (1.2)	–
	Forever Living Products	Use of Aloa Vera products (oral and topical)	1 (1.2)	–
	***Mind-body interventions***			
	Meditation	Undertaking a structured meditation exercise	8 (9)	6 (6)
	Guided imagery	Undertaking a guided imagery exercise with a therapist or self-administered	3 (3)	1 (1)
	Hypnosis	Undertaking a hypnotherapy session with a therapist	2 (2)	–
**Combined Interventions**	Yoga	Undertaking Yoga exercise	2 (2)	4 (4)

Participants could select more than one option. *Top five NPIs used by participants. # Top five NPIs participants plan to use.

For a large proportion of the participants (55%, n = 48), their caregivers decided if they should use an NPI, while 31% (n = 27) decided themselves. For 37% (n = 28), it was either recommended by healthcare professionals (e.g., physician, haematologist, nurse), a relative (e.g., grandmother), friends, or a teacher. A minority of participants reported changing their pain medications due to NPI use (12%, n = 15) and cited the effectiveness of NPIs as the reason for reduced intake of their pain medication. The analysis of associations revealed a significant relationship between reported sex and past use of NPIs (X^2^(1, N = 123) =9.2, p = .002), where females were more likely to have used an NPI. There was no significant relationship between past use of NPIs and other socio-demographic variables such as level of education (X^2^(2, N = 122) =1.7, p = .41) and religion (X^2^(2, N = 120) =.7, p = .72). The analysis of associations revealed significant relationships between various NPIs and socio-demographic variables (Table 4), such as past use of herbal products and frequency of SCC in the past 12 months (χ2 (2, N = 123) =5.6, p = .05), local heat application and age of initial diagnosis (χ2 (4, N = 123) =13.5, p = .009), local heat application and religion (χ2 (3, N = 123) =9.5, p = .008).

**Table 4:** Association Between Past Use of Specific Non-Pharmacological Interventions and Demographic Variables*.

Characteristics	Users, n (%)	Non-users, n (%)	Pearson chi-square value (df)	P value
**Herbal Products**				
**Frequency of SCC in last 12 months**				
≤5	18 (58)	66 (78)	5.6 (2)	0.05
6–10	12 (39)	15 (17)		
11 - 15	1 (3)	4 (5)		
**Local Heat**				
**Age of initial diagnosis**				
Birth	2 (10)	8(8)	13.5 (4)	0.009
0 - 5 years	5 (24)	50 (50)		
6 - 10 years	10 (48)	32 (32)		
11 - 15 years	2 (10)	11 (11)		
16 + years	2 (10)	0 (0)		
**Religion**				
Christianity	15 (68)	32 (33)	9.5 (2)	0.008
Islam	7 (32)	65 (66)		
Prefer not to say	0 (0)	1 (1)		
**Massage**				
**Religion**				
Christianity	25 (53)	22 (30)	6.8 (2)	0.03
Islam	22 (47)	50 (69)		
Prefer not to say	0 (0)	1 (1)		
**Prayer**				
**Age of initial diagnosis**				
Birth	1 (4)	9 (10)	11.2 (4)	0.02
0 - 5 years	8 (30)	47 (50)		
6 - 10 years	10 (37)	32 (34)		
11 - 15 years	7 (26)	6 (6)		
16 + years	1 (1)	1 (4)		
**Religion**				
Christianity	15 (56)	32 (34)	7.9 (2)	0.02
Islam	11 (41)	61 (66)		
Prefer not to say	1 (4)	0 (0)		
**Sleep/Rest**				
**Age**				
≤15 years	18 (90)	57 (55)	8.4 (1)	0.004
>15 years	2 (10)	46 (45)		

*Only significant results are included in this table; see (S3 File) for the complete subgroup analyses.

### Recent use of NPIs

**Fig 3:**
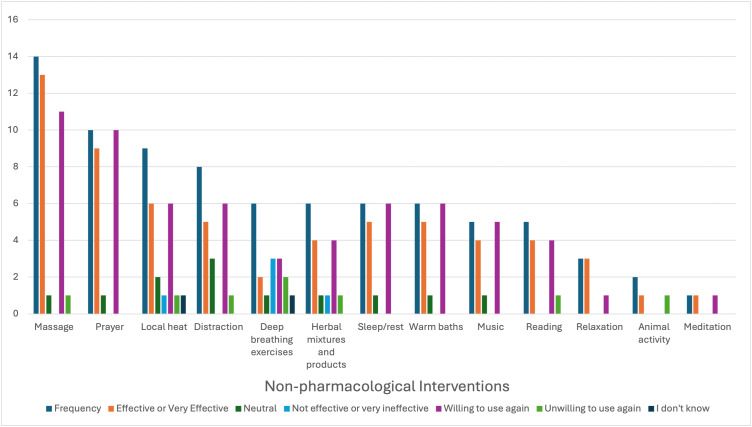
Recent use of NPIs: Perceived effectiveness of intervention and willingness to use again (n = 20).

**Fig 4:**
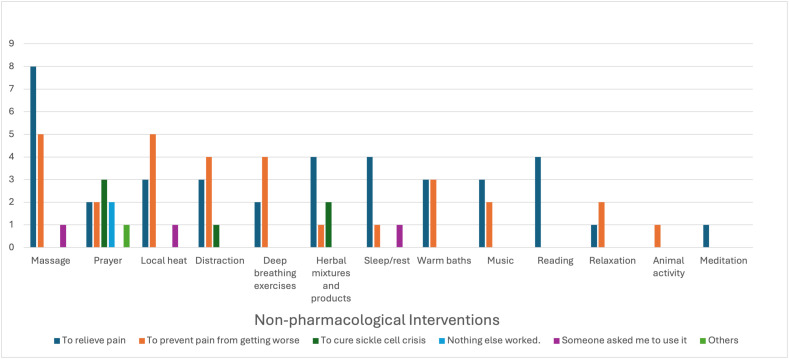
Recent use of NPIs: Reported reason for use (n = 20).

Some participants (16%, n = 20) indicated that they had used at least one NPI to manage pain during SCC in the last two weeks of completing the survey. The participants used 13 different NPIs (Fig 2). Most participants (70%, n = 14) used massage and found it very effective or effective (92.8%, n = 13), and 79% (11) planned to use it again. Figs 3 and 4 show all the NPIs, perceived effectiveness, willingness to use the NPI again, and the participants’ reasons for using these NPIs.

### Future use of NPIs

Slightly over half of the participants (52%, n = 64) planned to use NPIs in the future to manage SCC pain, while 30% (n = 37) were unsure. Participants who indicated they would not use NPIs in the future cited various reasons such as lack of knowledge about NPIs, their safety and effectiveness, lack of caregivers’ and healthcare providers’ support, the ineffectiveness of NPIs, and some felt NPIs were not a necessity for them. The most common NPIs that the participants planned to use in the future were herbal products (45%, n = 46), massage (33%, n = 34) and prayer (30%, n = 30). See Table 3 for the list of preferred NPIs for past and future use. There was no significant relationship between future use of NPIs and socio-demographic variables, including sex (X^2^(2, N = 116) = 1.3, p = .53), level of education (X^2^ (4, N = 115) = 1.1, p = .89) and religion (X^2^(4, N = 113) = 5.1, p = .27). The analysis of associations revealed significant relationships between various NPIs and socio-demographic variables (Table 5), such as local heat application and sex (χ2 (4, N = 123) =4.2, p = .04), massage and level of education (χ2 (2, N = 123) = 7.5, p = .02).

**Table 5:** Association Between Future Use of Specific Non-Pharmacological Interventions and Demographic Variables*.

Characteristics	Users, n (%)	Non-users, n (%)	Pearson chi-square value (df)	P value
**Local Heat**				
**Sex**				
Female	4 (25)	56 (52)	4.2 (1)	0.04
Male	12 (75)	51 (48)		
**Massage**				
**Level of Education**				
Primary	4 (67)	80 (73)	13.9 (2)	<.001
Secondary	0 (0)	27 (25)		
University/Polytechnic/College of Education	2 (33)	3 (3)		
**Meditation**				
**Frequency of SCC in last 12 months**				
≤5	25 (53)	22 (30)	6.8 (2)	0.03
6–10	22 (47)	50 (69)		
11 - 15	0 (0)	1 (1)		
**Religion**				
Christianity	6 (100)	41 (36)	9.9 (2)	0.007
Islam	0 (0)	72 (63)		
Prefer not to say	0 (0)	2 (2)		
**Prayer**				
**Frequency of SCC in last 12 months**				
≤5	25 (86)	59 (68)	10.8 (2)	0.004
6–10	1 (3)	26 (30)		
11 - 15	3 (10)	2 (2)		
**Age of initial diagnosis**				
Birth	0 (0)	10 (11)	9.2 (4)	0.05
0 - 5 years	9 (31)	46 (50)		
6 - 10 years	15 (52)	27 (29)		
11 - 15 years	4 (14)	9 (10)		
16 + years	1 (3)	1 (1)		
**Religion**				
Christianity	21 (70)	26 (29)	16.4 (2)	<0.001
Islam	9 (30)	68 (69)		
Prefer not to say	2 (2)	0 (0)		
**Reading**				
**Frequency of SCC in last 12 months**				
≤5	7 (70)	77 (73)	7.1 (2)	0.03
6–10	1 (10)	26 (25)		
11 - 15	2 (20)	3 (3)		
**Relaxation**				
**Occupation**				
Student	5 (83)	114 (98)	5.3 (1)	0.02
Others	1 (17)	2 (2)		
**Sleep/rest**				
**Occupation**				
Student	19 (91)	100 (99)	5.3 (1)	0.02
Others	2 (9)	1 (1)		

*Only significant results are included in this table; see (S3 File) for the complete subgroup analyses.

### Difficulty in using NPIs

Most participants (59%, n = 73) reported no difficulty using NPIs during SCC. There was a significant relationship between sex and difficulty using NPIs (X^2^ (1, N = 86) = 5, p = .02), with males being more likely to have difficulty using NPIs. There was no significant relationship between difficulty using NPIs and other socio-demographic variables such as level of education (X^2^(2, N = 85) = 2.4, p = .3) and religion (X^2^(2, N = 84) =.2, p = .9).

### Qualitative analysis

Seven key themes emerged from the analysis of the open-ended question responses. The open-ended questions focused on the challenges of using NPIs at home and in hospitals.

### Healthcare provider and caregiver disapproval

Several participants (n = 25) suggested that healthcare professionals (such as doctors and nurses) and caregivers sometimes disapproved of NPI use and that using NPI would lead to their displeasure. For example, one participant (a 16-year-old male who used deep breathing exercises, local heat, massage, and prayer) wrote, “Hospital staff will complain,” implying this was one perceived barrier to NPI uptake.

### SCC pain limits the practical use of NPIs

A number of participants (n = 16) reported that the severity of the pain during SCC made it very challenging to use NPIs in practice.

### Limited access to NPIs

Participants (n = 16) also cited challenges that limited their access to NPIs, such as unstable electricity supply, having to travel long distances to use them, or scarcity of specific NPIs (e.g., unavailability of Akpuru leaves [Ficus capensis] locally), lack of funds, and/or poor internet access. One 15-year-old who used several NPIs, including distraction (games), local heat, and massage, indicated there was a “lack of electricity to play games.” Another participant, an 18-year-old female who used various NPIs, including distraction (videos), herbal products and massage, also reported a “Lack of strong internet connection when watching movies”.

### Lack of knowledge and misconceptions about NPIs

Some participants (n = 13) indicated they lacked knowledge about effective and safe NPIs, while others reported that NPIs should not be used in conjunction with medical treatment. One 14-year-old male participant, who used distraction (games), herbal products, massage, and sleep/rest, wrote, “[It] cannot be used while receiving treatment in hospital; it was just an alternative.”.

### Ineffectiveness or side-effect of the NPI

A few participants (n = 4) reported that certain NPIs were ineffective in relieving their pains, had side effects such as vomiting and had a bitter taste, specifically herbal products. One 18-year-old male who reported using herbal products stated, “I vomit when I use it.”

### Other challenges associated with NPI use

A minority of participants reported other difficulties associated with NPI use, such as environmental disturbances (noise) in hospitals (n = 3) and lack of access to preferred NPIs in hospitals (n = 2).

### Preferred medium for NPI educational resources

**Fig 5:**
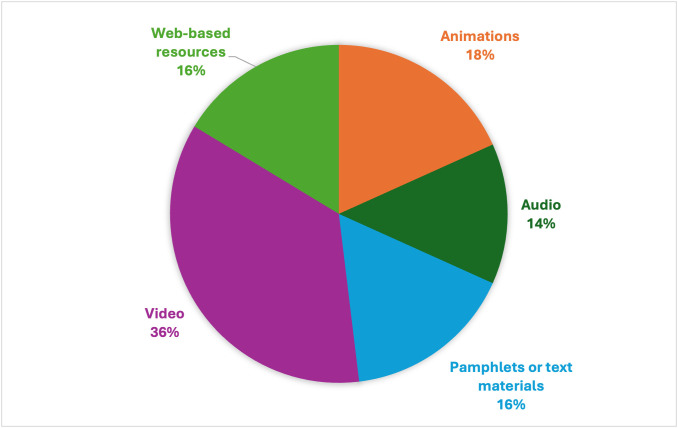
Preferred medium of receiving NPI resources (n = 104).

In answering the question exploring how the participants would like to receive information and education on NPI use, a large proportion of participants indicated they would prefer videos (36%, n = 37), followed by animations (18%, n = 19) as their preferred medium for dissemination (Fig 5).

## Discussion

The study revealed that the majority of adolescents employed NPIs, with 8 out of 10 reporting using at least one NPI. This finding is consistent with other studies conducted in Nigeria and higher-income countries, such as the US. One study investigating SCC pain management in paediatric patients in a hospital in South-West Nigeria found that 100% of participants utilized prayer as an NPI during emergency admission for SCC [23]. In another Nigerian study, Oshikoya et al. [24] reported that only 36% of their participants had used any NPI. However, that study was not specific to SCC but to overall SCD management. In the US, a study reported a 63% prevalence rate of NPI use among pediatric patients with SCD [37]. A systematic review of 24 studies reported a prevalence rate of NPI use among SCD patients ranging from 36 to 84.5% [38]. In the current study, the participants’ main reported reasons for NPI use were ‘to feel better’ and ‘to prevent the pain from getting worse.’ This is not surprising considering the severity of SCC pain, patients’ reports of inadequate pain management and the stigmatization adolescents with SCD often experience whenever they request more analgesia [39]. Given these challenges, it is understandable that many adolescents may seek alternative/complementary interventions to relieve their pain, including potentially harmful ones, such as herbal compounds with unknown contents.

Participants reported using a wide range of NPIs, and the top five interventions were massage, herbal products and mixtures, prayer, local heat and sleep/rest. Another study conducted in Nigeria reported prayer and fluid intake as the only NPIs used for SCC in children and adolescents during emergency admission, while a study conducted in the US reported massage, heat application, fluid intake, prayer and rest as commonly used NPIs by caregivers of children and adolescents with SCD [40]. In contrast to our findings, survey-based studies conducted in the US have reported sleeping, watching TV, talking with people and playing video games as the most common NPIs used by adolescents [41,42]. There is a contrast in the types of widely used NPIs between adolescents in Nigeria and the US. This might be due to cultural differences and resource availability, such as constant electricity. However, studies focusing on general SCD pain management in children and adolescents in the US have identified prayer, massage and herbal medicines as commonly being used NPIs [37,43]. These findings suggest these types of NPIs are not limited to Nigerian adolescents, but they are frequently used in the US and may be among adolescents in other parts of the world. This is significant because prayer and use of herbal products/mixtures, two common forms of NPIs, are not recommended by experts [20,21]. Whilst prayer is a low-risk intervention, as a faith-based non-specific practice, establishing efficacy is problematic. Herbal products also present significant risks due to low-quality control of the dosage, uncertainty over the actual contents of the remedy being used, and insufficient scientific evidence regarding their safety and effectiveness. Therefore, the potential hazards of these herbal products on SCC pain and SCD outcomes might not only impact Nigerians and Americans but could be an ongoing impediment to achieving optimal SCD patient outcomes worldwide.

The most frequently used NPIs fall under biomedical interventions. Within this category, the most prevalent interventions involved massage and local heat application, which are widely recommended by experts in SCC pain management [20,21]. Also, among recent NPI users, massage was the most frequently used, with 93% reporting it as effective and 79% planning to use it again. A retrospective study investigating massage therapy sessions in a U.S. clinic reported that massage led to significant reductions in SCC pain, stress, and anxiety in children and adolescents, with a majority of the participants reporting pain reductions meeting clinically significant thresholds and no adverse effects [44]. In that study, massage was provided by a trained massage therapist. In this current study, it is unclear who provided massages for the participants. Also, some adolescents reported using substances such as Robb (a Nigerian product), Palm Kernel Oil, Olive Oil and Shea Butter to massage the affected areas. However, it is unclear whether adolescents and/or caregivers received any training on massage or whether these techniques were traditionally developed. It is important to further explore these practices and the learning needs of adolescents and their caregivers, in order to develop appropriate educational resources to promote safe and effective massage practices during SCC.

Participants reported herbal and nutritional interventions as the second most common form of NPI used, with herbal products being the most widely used NPI within this category. Interestingly, 66% of recent NPI users found herbal products to be effective and expressed willingness to use them again. It is noteworthy that a large proportion of adolescents reported being inclined to consider herbal products as their choice for future NPI use. This trend is concerning due to the limited evidence of the safety and efficacy of these products. However, Ameh et al. [45] argue that before Dr James Herrick discovered SCD in 1910, Africans had been using herbal products to treat SCD and its symptoms for centuries. Despite this historical context, the high mortality rate associated with SCD before the 21st century raises questions about the effectiveness and safety of such practices [46].

Nevertheless, given herbal products have a long history of traditional use, and may be effective in helping treat some SCD symptoms such as pain, further exploration of their value is appropriate [45]. For example, a recent systematic review documented the use of herbal products in four African countries and reported some had anti-sickling properties [47]. Since the 1970s, the properties and mechanisms of action of some of these herbs have been studied in Nigerian universities as potential pharmacological products. This research eventually led to the development of the drug Niprisan, (a drug that prevents the polymerization of sickle hemoglobin) reducing the frequency of SCC without adverse effects, unlike existing hydroxyurea [45]. The efficacy and safety of Niprisan have been evaluated through various clinical trials, and the drug was approved and commercialized in Nigeria till the production company closed down due to mismanagement [48–50].

Currently, it remains unclear which specific types of herbal products are most commonly used by adolescents with SCD. Nigeria’s National Agency for Food and Drug Administration and Control (NAFDAC) recently cautioned against the widespread distribution of unapproved and potentially hazardous herbal products [51]. Moreover, it is common for individuals to concoct their own herbal mixtures with limited knowledge of the properties of the substances involved or their interactive effects. Future studies should aim to identify the specific types of herbal products adolescents use and develop appropriate educational resources.

A reliance on the supernatural to alleviate SCC pain was also highlighted in the survey, as prayer was the third most used NPI and the third top NPI adolescents plan to use. Of the adolescents who prayed during a recent SCC, 90% described it as effective, and 100% were willing to use it again. This is not a surprising finding as Nigeria has often been described as a highly religious country with 50% Muslim and 48% Christian populations [52]. Moreover, for many centuries, SCD was regarded as a spiritual or paranormal affliction, and these beliefs are still prevalent in Nigeria today [45]. The severity of SCC, sub-optimal pain management strategies, and the overwhelming nature of the pain can render adolescents feeling helpless, and hence, they resort to faith-based interventions. The use of prayer would appear to have a calming psychological effect. Despite the limited number of studies on the effects of prayer in adolescents during SCC, one US study of children (5–10 years) reported that prayer comforted them. They described specific ways prayer helped them when they had SCC, such as feeling that God was hugging, smiling and healing them [53]. Although patients report some comfort from the use of prayer, the use of spiritually focused NPIs would seem highly population-specific. How such approaches and specific techniques might be used by different religious denominations or their value for non-religious groups has yet to be determined.

Furthermore, as widely recognized, cultural beliefs and practices influence pain perception, expression and management [54,55]. The common use of spiritual and herbal interventions may be rooted in Nigerian culture, which includes spiritual practices and traditional healing. Societal beliefs regarding pain expression might also influence the NPI types of adolescents’ use. Developing culturally tailored interventions that reflect the societal values and beliefs can promote the uptake of evidence-based interventions [54].

The participants in this study highlighted various factors that influenced their ability to use NPIs to manage SCC pain. One significant factor was the reported disapproval of some NPI use by healthcare providers and caregivers. Since healthcare providers and caregivers are key stakeholders in adolescents’ SCD care, their opinions on interventions influence the adolescents’ usage. According to the participants, most of them were directed to use an NPI by their caregivers, while a few were directed by healthcare providers. Some comments from the participants suggested that healthcare providers do not believe in the value of NPIs for managing SCC, although it was unclear which NPIs they approved or disapproved of. This lack of trust may be due to the limited evidence supporting the efficacy of most NPIs in SCC management [22]. Furthermore, healthcare providers’ disapproval might support caregivers to discourage their adolescents from using NPIs. It is imperative to explore further the perspectives of caregivers and healthcare providers on NPI use in managing SCC pain and to address their learning needs regarding NPIs. Seeking their opinions on specific NPIs, such as distraction and massage, which have been widely recommended by experts for SCC pain management, is also important.

One significant factor is the severity of the pain. SCC pain is excruciating, making it difficult for adolescents to engage with many NPIs, such as sleep/rest and distraction. This emphasizes the importance of an integrative approach, where pharmacological interventions are used alongside NPIs. Intriguingly, about 12% of the participants reported discontinuing their use of analgesics because of the effectiveness of the NPI. While this outcome may be desirable, adolescents should be educated to adopt an integrative, multimodal approach when experiencing severe pain and only discontinue analgesics when they do not add value to their pain management.

Interestingly, the past use of NPIs and the difficulty associated with their use appeared to be sex-specific. Females reported being more likely to have used an NPI, and males were more likely to have difficulty using an NPI. These findings might be related to the suggested sex differences in SCC severity [56]. There are conflicting findings regarding the nature of these sex differences. A study found that females have high pain intensity, more SCC episodes, and more hospital admissions in a year [56]. This finding might explain why females are using NPIs more than males. However, other studies report that males have more SCC episodes and more hospital admissions in a year [57,58]. The basis for these sex differences remains unclear. Future studies should further explore the sex and gender differences related to NPI use and the associated difficulty with their use. Based on the analysis of association, the age of initial diagnosis appears to influence the use of prayer and local heat. SCC frequency influenced the use of herbal products and the future use of reading, meditation and prayer. These findings may be related to more prolonged and frequent exposure to SCC, and continuous efforts to identify the most effective NPIs to reduce SCC pain. Religion appears to influence the use of massage, local heat, and prayer, as well as the future use of meditation and prayer. These results are understandable as Christianity and Islam encourage praying for healing from diseases, and meditating on Biblical and Quranic scriptures for healing, whilst non-religious people might not seek healing through prayers [59,60]. Moreover, in religions such as Christianity, healing is associated with the laying of hands over the affected parts; these beliefs might explain the relationship between religion and massage [61].

In exploring the preferred medium for receiving educational resources on NPIs, the participants preferred videos followed by animations and pamphlets. Nigeria spans a vast, dispersed geographical area, encompassing 36 states and a Federal Capital Territory, over 923,768 km². Therefore, it is essential to choose a medium that can be easily accessed across the rural and urban areas of the country. Although using digital media over the Internet would seem the obvious medium for widespread dissemination, web-based resources were among the least preferred options, possibly due to accessibility issues. How can videos and animations be widely distributed across Nigeria? One option is to distribute the videos on DVDs to adolescents through sickle cell clubs, foundations, and clinics nationwide. However, it is unclear how many Nigerians own a DVD player or whether this would be a practical medium considering the frequent power outages in the country. Alternatively, videos could be played in clinics nationwide; however, frequent power outages might also affect their delivery. Pamphlets and other text-based materials would be suitable media as they do not require electricity, but distributing them across the country would be relatively expensive.

Techniques such as massage and guided imagery might be better demonstrated in a video rather than described in a pamphlet. In Nigeria, similar educational resources have been delivered in person [62–64] through mobile health intervention [65] and pamphlets [66]. While these methods were found feasible in various randomized controlled trials, it is unclear if they were sustainable long-term. Given the country’s large population, utilizing multiple media hosted on a website might be beneficial. Though Sasu [67] reported that 27% of the Nigerian population uses a smartphone, and internet access has improved in Nigeria, internet connectivity and electricity supply challenges might affect their use. For instance, in this study, paper-based surveys were preferable to online-based surveys due to the limited access to smartphones and the Internet. These challenges associated with disseminating educational resources are not limited to Nigeria alone, as other LMICs also experience similar challenges [68].

## Strengths and limitations

This is the first study to explore a significant issue that is impacting SCC pain care in adolescents and provides insights into the various types of NPIs used by adolescents in Nigeria. This study provides unique insights into NPI use in an LMIC, as most studies on NPIs are conducted in high-income countries [22]. Other strengths of this study include the use of an age-appropriate survey developed in conjunction with experts and obtaining adolescents’ perspectives from more than 50% of the states in Nigeria. However, the small sample size limits the generalization of the findings across Nigeria. Moreover, the paper-based survey was disseminated in only six states; hence, those with limited Internet access in the remaining 31 states did not have the opportunity to participate. Therefore, future studies should target these populations. Additionally, longitudinal studies may help identify changes in adolescents’ use of NPIs over time. The open-ended survey responses were coded by one author. Lastly, while efforts were made to ensure the quality of the online survey data, the data could have been biased as the participants’ identities could not be verified to ensure their eligibility. The possibility of fraudulent entries was minimized by applying preventive measures such as using a reCAPTCHA (Completely Automated Public Turing test to tell Computers and Humans Apart) to identify and prevent bots from completing the survey and by enabling the “prevent multiple submissions, bot detection and security scan monitor” settings in Qualtrics [69].

## Conclusion

This study provides the first evidence that Nigerian adolescents use various NPIs to manage their pain during SCC, including traditional remedies along with physical and spiritual interventions. As reported by participants, some healthcare providers and caregivers oppose the use of NPIs, but it is unclear if this is limited to only questionable interventions. The findings highlight the need to educate adolescents on safe and effective NPIs and raise caution on the use of questionable NPIs such as herbal products. To provide this education, adopting a sustainable approach to disseminating these educational resources in Nigeria is crucial and remains to be further investigated.

This study demonstrates that pain severity during SCC is the key motivator for adolescents to use various NPIs, and these interventions are widely used regardless of healthcare providers’ opinions. Many adolescents perceived many NPIs as effective. Hence, healthcare providers should seek patients’ broader perspectives on their SCC pain management and the effectiveness of their treatment plan and incorporate appropriate NPIs into their treatment plans. Current policies in Nigeria on SCC pain management should be reviewed and as new evidence arises, updated to include effective NPIs. Of all the NPIs reportedly used, none have high-quality scientific evidence supporting their use, and only massage, heat application, and distraction were recommended by experts in SCC pain management [20,21]. However, the widespread use of herbal products and other NPIs with limited scientific evidence of their safety or effects on SCD outcomes is evident. This warrants the attention of clinicians and researchers as there is an urgent need to further investigate specific NPI use and their safety and impact on SCD outcomes. Particularly, researchers, pharmaceutical companies, and funding agencies could prioritize research on the most commonly used herbal products, as there seems to be a high demand for them. It is also important for researchers to further investigate NPI uptake for SCC in LMICs as these are the primary geographical locations of SCD patients, and NPI uptake differs from that in high-income countries. Similarly, appropriate forms of education and dissemination for NPI use need to be considered with respect to available information, communications infrastructure, and user preferences.

Furthermore, these research findings imply the need for a cultural shift from heavy reliance on pharmacological management to an integrative pain management approach. This approach can better address the multidimensional nature of SCC pain and lead to better pain outcomes.

## Supporting information

S1 File Questionnaire. (PDF)

S2 File Inclusivity in global research. (DOCX)

S3 File Subgroup analyses. (DOCX)
