# Supplementary material for: Adolescents’ perspectives on non-pharmacological pain interventions for sickle cell crisis management: A population-based survey
Source: PLoS One. 2025 Aug 19;20(8):e0330127. doi: 10.1371/journal.pone.0330127 (PMC12364358; doi:10.1371/journal.pone.0330127)
Supplement: S1 File — (PDF) [file pone.0330127.s001.pdf]

Questionnaire

Demographic and Health Information Questionnaire

Directions: Please answer the following questions.

Socio-demographic Information

1. Age in years ………………………………………………
2. Sex
   - ☐Male
   - ☐Female
   - ☐Prefer not to say
3. Level of education
   - ☐none
   - ☐primary
   - ☐secondary/high school
   - ☐university/polytechnic/college of education
4. Occupation
   - ☐student
   - ☐Others (specify) ……………………………
5. State of residence………………..
6. Religion…………………………

Health information

7. At what age were you and/or your parents first told you had sickle cell disease?
   - ☐Birth
   - ☐0-5 years
   - ☐6-10 years
   - ☐11-15 years
   - ☐16+ years

8. Do you know the type of sickle cell disease you have (genotype)?
   - ☐Sickle cell anaemia (SS)
   - ☐Sickle haemoglobin C (SC)
   - ☐Don't know
   - ☐Others (specify)……………………..

9. How many times have you had crises in the last 12 months?.............

10. When you had these crises, how many times did you go to the hospital to see the doctor?...............

11. When you had these crises, how many times did you or your parents treat it at home?............

12. How bad was your pain on average in last crisis you had?

☐	☐	☐	☐	☐	☐	☐	☐	☐	☐	☐
0	1	2	3	4	5	6	7	8	9	10
**No pain**										**Worst pain you can think of**

Non-Pharmacological Pain Management Strategies Questionnaire

In this section, we will ask you questions about other things you use to stop pain when you have crises. We will call these other things, METHODS. Example of these methods include:
Herbal mixture, herbs in capsules or creams, vitamins, minerals, special food, juices, or teas, hypnosis, imagery, prayer, relaxation, mindfulness, acupuncture, massage, traditional bone setting, osteopathy, chiropractic, yoga, aromatherapy, copper bracelets, magnets, reflexology and body scarification.

Section A: Past use of other methods aside drugs

13. Have you <u>ever</u> used any of these methods to reduce your pain during a sickle cell crisis?

- ☐ Yes. Why? (Tick all that apply)
  - ☐ To feel better.
  - ☐ To prevent pain from getting worse.
  - ☐ To cure sickle cell crisis.
  - ☐ It is natural/safe.
  - ☐ Nothing else worked.
  - ☐ It was prescribed.
  - ☐ Other, *please specify:* ____________________
- ☐ No. Why not? (Tick all that apply)
  - ☐ I believed it would not reduce my pain.
  - ☐ I did not have enough information about it.
  - ☐ The health care team did not prescribe it.
  - ☐ I did not want to use it. Why? ____________________
  - ☐ It costs too much.
  - ☐ It was difficult to find.
  - ☐ I was afraid of the side effects or mixing it with my medical treatment.
  - ☐ Others, *please specify*: ____________________

If your answer to Question 14 is No, move to Question 18

14. Which of these methods did you use? (Tick all that apply)

- ☐ Deep breathing exercises
- ☐ Distraction
- ☐ Guided imagery
- ☐ Heat application
- ☐ Herbal products
- ☐ Hypnosis
- ☐ Massage
- ☐ Music
- ☐ Meditation
- ☐ Play with pet
- ☐ Play with pet
- ☐ Prayer
- ☐ Reading
- ☐ Recreational activities
- ☐ Progressive muscle relaxation
- ☐ Relaxation
- ☐ Sleep/rest
- ☐ Virtual reality
- ☐ Warm bath
- ☐ Yoga

Others, *please specify*: ______________________________

15. **Who decided that you should use these methods?** (Tick all that apply)

- ☐ Me
- ☐ My parents
- ☐ A doctor, nurse, or someone else who works in the hospital. Who? ____________________________
- ☐ Other providers (for example: herbalist, acupuncturist, Pastor, and Imam). Who?____________________
- ☐ Someone else. Who? ____

16. Have you changed how you use your drugs because you used these methods?

☐ Yes.

How?

Why?

☐ No, I did not change my medical treatment

Section B: Recent use of other methods aside drugs.

17. **Have you used any of these methods in the past two weeks?** ☐ Yes ☐ No
    If no, move to Question 19.
18. **If yes, please tick the methods you used below. Also, answer the following questions for each method. Tick all the ones you have used.**

☐ **Deep breathing exercises**

Why did you use it?
- ☐ To relieve pain.
- ☐ To prevent pain from getting worse.
- ☐ To cure sickle cell crisis.
- ☐ Nothing else worked.
- ☐ A nurse/doctor asked me to use it.
- ☐ Someone else aside my nurse or doctor asked me to use it. Who?__________________
- ☐ If others, please specify___________________

Was it effective in reducing pain during crisis?
- ☐ Very effective
- ☐ Effective
- ☐ Neutral
- ☐ Not effective
- ☐ Very ineffective

Are you willing to use it again?
If yes, why?_____________________
If no, why?______________________
- ☐ I don't know

☐ **Distraction (E.g.: Games, blowing bubbles and video such as comedy skits and movies)**

Which type(s) of distraction did you use?____.

Why did you use it?
- ☐ To relieve pain.
- ☐ To prevent pain from getting worse.
- ☐ To cure sickle cell crisis.
- ☐ Nothing else worked.
- ☐ A nurse/doctor asked me to use it.
- ☐ Someone else aside my nurse or doctor asked me to use it. Who?__________________
- ☐ If others, please specify___________________

Was it effective in reducing pain during crisis?
- ☐ Very effective
- ☐ Effective
- ☐ Neutral
- ☐ Ineffective
- ☐ Very ineffective

Are you willing to use it again?
If yes, why?_____________________
If no, why?______________________
- ☐ I don't know

☐ **Guided Imagery**

Why did you use it?
- ☐ To relieve pain.
- ☐ To prevent pain from getting worse.
- ☐ To cure sickle cell crisis.
- ☐ Nothing else worked.
- ☐ A nurse/doctor asked me to use it.
- ☐ Someone else aside my nurse or doctor asked me to use it. Who?__________________
- ☐ If others, please specify___________________

Was it effective in reducing pain during crisis?
- ☐ Very effective
- ☐ Effective
- ☐ Neutral
- ☐ Ineffective
- ☐ Very ineffective

Are you willing to use it again?
If yes, why?_____________________
If no, why?______________________
- ☐ I don't know

☐ **Heat application**

Why did you use it?
- ☐ To relieve pain.
- ☐ To prevent pain from getting worse.
- ☐ To cure sickle cell crisis.
- ☐ Nothing else worked.
- ☐ A nurse/doctor asked me to use it.
- ☐ Someone else aside my nurse or doctor asked me to use it. Who?__________________
- ☐ If others, please specify___________________

Was it effective in reducing pain during crisis?
- ☐ Very effective
- ☐ Effective
- ☐ Neutral
- ☐ Ineffective
- ☐ Very ineffective

Are you willing to use it again?
If yes, why?_____________________
If no, why?______________________
- ☐ I don't know

☐ **Herbal products**

Why did you use it?

- ☐ To relieve pain.
- ☐ To prevent pain from getting worse.
- ☐ To cure sickle cell crisis.
- ☐ Nothing else worked.
- ☐ A nurse/doctor asked me to use it.
- ☐ Someone else aside my nurse or doctor asked me to use it. Who?__________________
- ☐ If others, please specify___________________

Was it effective in reducing pain during crisis?

- ☐ Very effective
- ☐ Effective
- ☐ Neutral
- ☐ Not effective
- ☐ Very ineffective

Are you willing to use it again?
If yes, why?_____________________
If no, why?______________________

- ☐ I don't know

☐ **Massage**

Why did you use it?

- ☐ To relieve pain.
- ☐ To prevent pain from getting worse.
- ☐ To cure sickle cell crisis.
- ☐ Nothing else worked.
- ☐ A nurse/doctor asked me to use it.
- ☐ Someone else aside my nurse or doctor asked me to use it. Who?__________________
- ☐ If others, please specify___________________

Was it effective in reducing pain during crisis?

- ☐ Very effective
- ☐ Effective
- ☐ Neutral
- ☐ Ineffective
- ☐ Very ineffective

Are you willing to use it again?
If yes, why?_____________________
If no, why?______________________

- ☐ I don't know

☐ **Meditation**

Why did you use it?

- ☐ To relieve pain.
- ☐ To prevent pain from getting worse.
- ☐ To cure sickle cell crisis.
- ☐ Nothing else worked.
- ☐ A nurse/doctor asked me to use it.
- ☐ Someone else aside my nurse or doctor asked me to use it. Who?__________________
- ☐ If others, please specify___________________

Was it effective in reducing pain during crisis?

- ☐ Very effective
- ☐ Effective
- ☐ Neutral
- ☐ Ineffective
- ☐ Very ineffective

Are you willing to use it again?
If yes, why?_____________________
If no, why?______________________

- ☐ I don't know

☐ **Music**

Why did you use it?

- ☐ To relieve pain.
- ☐ To prevent pain from getting worse.
- ☐ To cure sickle cell crisis.
- ☐ Nothing else worked.
- ☐ A nurse/doctor asked me to use it.
- ☐ Someone else aside my nurse or doctor asked me to use it. Who?__________________
- ☐ If others, please specify___________________

Was it effective in reducing pain during crisis?

- ☐ Very effective
- ☐ Effective
- ☐ Neutral
- ☐ Ineffective
- ☐ Very ineffective

Are you willing to use it again?
If yes, why?_____________________
If no, why?______________________

- ☐ I don't know

☐ **Playing with pets**

Why did you use it?

☐ To relieve pain.
☐ To prevent pain from getting worse.
☐ To cure sickle cell crisis.
☐ Nothing else worked.
☐ A nurse/doctor asked me to use it.
☐ Someone else aside my nurse or doctor asked me to use it. Who?__________________
☐ If others, please specify___________________

Was it effective in reducing pain during crisis?

☐ Very effective
☐ Effective
☐ Neutral
☐ Ineffective
☐ Very ineffective

Are you willing to use it again?

If yes, why?_____________________

If no, why?______________________

☐ I don't know

☐ **Progressive Muscle Relaxation**

Why did you use it?

☐ To relieve pain.
☐ To prevent pain from getting worse.
☐ To cure sickle cell crisis.
☐ Nothing else worked.
☐ A nurse/doctor asked me to use it.
☐ Someone else aside my nurse or doctor asked me to use it. Who?__________________
☐ If others, please specify___________________

Was it effective in reducing pain during crisis?

☐ Very effective
☐ Effective
☐ Neutral
☐ Ineffective
☐ Very ineffective

Are you willing to use it again?

If yes, why?_____________________

If no, why?______________________

☐ I don't know

☐ **Prayer**

Why did you use it?

☐ To relieve pain.
☐ To prevent pain from getting worse.
☐ To cure sickle cell crisis.
☐ Nothing else worked.
☐ A nurse/doctor asked me to use it.
☐ Someone else aside my nurse or doctor asked me to use it. Who?__________________
☐ If others, please specify___________________

Was it effective in reducing pain during crisis?

☐ Very effective
☐ Effective
☐ Neutral
☐ Ineffective
☐ Very ineffective

Are you willing to use it again?

If yes, why?_____________________

If no, why?______________________

☐ I don't know

☐ **Recreational activities**

Why did you use it?

☐ To relieve pain.
☐ To prevent pain from getting worse.
☐ To cure sickle cell crisis.
☐ Nothing else worked.
☐ A nurse/doctor asked me to use it.
☐ Someone else aside my nurse or doctor asked me to use it. Who?__________________
☐ If others, please specify___________________

Was it effective in reducing pain during crisis?

☐ Very effective
☐ Effective
☐ Neutral
☐ Ineffective
☐ Very ineffective

Are you willing to use it again?

If yes, why?_____________________

If no, why?______________________

☐ I don't know

☐ **Relaxation**
Why did you use it?
- ☐ To relieve pain.
- ☐ To prevent pain from getting worse.
- ☐ To cure sickle cell crisis.
- ☐ Nothing else worked.
- ☐ A nurse/doctor asked me to use it.
- ☐ Someone else aside my nurse or doctor asked me to use it. Who?__________________
- ☐ If others, please specify___________________

Was it effective in reducing pain during crisis?
- ☐ Very effective
- ☐ Effective
- ☐ Neutral
- ☐ Ineffective
- ☐ Very ineffective

Are you willing to use it again?
If yes, why?_____________________
If no, why?______________________
- ☐ I don't know

☐ **Rest or Sleep**
Why did you use it?
- ☐ To relieve pain.
- ☐ To prevent pain from getting worse.
- ☐ To cure sickle cell crisis.
- ☐ Nothing else worked.
- ☐ A nurse/doctor asked me to use it.
- ☐ Someone else aside my nurse or doctor asked me to use it. Who?__________________
- ☐ If others, please specify___________________

Was it effective in reducing pain during crisis?
- ☐ Very effective
- ☐ Effective
- ☐ Neutral
- ☐ Ineffective
- ☐ Very ineffective

Are you willing to use it again?
If yes, why?_____________________
If no, why?______________________
- ☐ I don't know

☐ **Virtual reality**
Why did you use it?
- ☐ To relieve pain.
- ☐ To prevent pain from getting worse.
- ☐ To cure sickle cell crisis.
- ☐ Nothing else worked.
- ☐ A nurse/doctor asked me to use it.
- ☐ Someone else aside my nurse or doctor asked me to use it. Who?__________________
- ☐ If others, please specify___________________

Was it effective in reducing pain during crisis?
- ☐ Very effective
- ☐ Effective
- ☐ Neutral
- ☐ Ineffective
- ☐ Very ineffective

Are you willing to use it again?
If yes, why?_____________________
If no, why?____________________
- ☐ I don't know

☐ **Yoga**
Why did you use it?
- ☐ To relieve pain.
- ☐ To prevent pain from getting worse.
- ☐ To cure sickle cell crisis.
- ☐ Nothing else worked.
- ☐ A nurse/doctor asked me to use it.
- ☐ Someone else aside my nurse or doctor asked me to use it. Who?__________________
- ☐ If others, please specify___________________

Was it effective in reducing pain during crisis?
- ☐ Very effective
- ☐ Effective
- ☐ Neutral
- ☐ Ineffective
- ☐ Very ineffective

Are you willing to use it again?
If yes, why?_____________________
If no, why?_____________________
- ☐ I don't know

☐ **Others, please specify_____**

Why did you use it?

☐ To relieve pain.
☐ To prevent pain from getting worse.
☐ To cure sickle cell crisis.
☐ Nothing else worked.
☐ A nurse/doctor asked me to use it.
☐ Someone else aside my nurse or doctor asked me to use it. Who?__________________
☐ If others, please specify___________________

Was it effective in reducing pain during crisis?

☐ Very effective
☐ Effective
☐ Neutral
☐ Ineffective
☐ Very ineffective

Are you willing to use it again?

If yes, why?_____________________

If no, why?______________________

☐ I don't know

☐ **Others, please specify_____**

Why did you use it?

☐ To relieve pain.
☐ To prevent pain from getting worse.
☐ To cure sickle cell crisis.
☐ Nothing else worked.
☐ A nurse/doctor asked me to use it.
☐ Someone else aside my nurse or doctor asked me to use it. Who?__________________
☐ If others, please specify___________________

Was it effective in reducing pain during crisis?

☐ Very effective
☐ Effective
☐ Neutral
☐ Ineffective
☐ Very ineffective

Are you willing to use it again?

If yes, why?_____________________

If no, why?______________________

☐ I don't know

Section C: Challenges of using these methods for sickle cell crisis

19. Do you find it difficult to use any of these methods during a crisis?

☐ Yes, why?

☐ No

20. What problems have stopped you from using any of these methods during a crisis?

21. What problems have stopped you from using any of these methods during a crisis in the hospital?

22. What problems have stopped you from using any of these methods during a crisis at home?

Section D: Future use of other methods aside drugs

23. Do you plan on using any non-medication pain-relief methods in the future?

☐ Yes. (Go to question 23)
☐ No. Why not? ____
☐ Not sure

24. Which of the following methods would you like to use? (Check all that applies)

☐ Deep breathing exercises
☐ Distraction
☐ Guided imagery
☐ Heat application
☐ Herbal products
☐ Hypnosis
☐ Massage
☐ Music
☐ Meditation
☐ Play with pet
☐ Play with pet
☐ Prayer
☐ Reading
☐ Recreational activities
☐ Progressive muscle relaxation
☐ Relaxation delivered through virtual reality
☐ Sleep/rest
☐ Virtual reality
☐ Warm bath
☐ Yoga

Others, *please specify*: ____

25. How would you like to receive information on various methods that you can use to reduce your pain during a sickle cell crisis?

☐ Animations
☐ Audio
☐ Pamphlets or text materials
☐ Video
☐ Web-based resources

26. **Please provide any additional information about your treatments or the methods you use**____________

____

27. Do you have any additional comments?

____

____

Thank you for completing this questionnaire!
