# Supplementary material for: Adolescents’ perspectives on non-pharmacological pain interventions for sickle cell crisis management: A population-based survey
Source: PLoS One. 2025 Aug 19;20(8):e0330127. doi: 10.1371/journal.pone.0330127 (PMC12364358; doi:10.1371/journal.pone.0330127)
Supplement: S3 File — (DOCX) [file pone.0330127.s003.docx]

| **Characteristics** Deep-breathing exercises | **Users, n (%)** | **Non-users, n (%)** | **Pearson chi-square value (df)** | **P value** |
| --- | --- | --- | --- | --- |
|  | **Past Use** | | | |
| **Age** |  |  |  |  |
| ≤15 years | 5 (45) | 70 (63) | 1.2 (1) | 0.2 |
| >15 years | 6 (55) | 42 (37) |  |  |
| **Sex** |  |  |  |  |
| Male | 6 (55) | 54 (48) | 0.16 (1) | 0.7 |
| Female | 5 (45) | 58 (52) |  |  |
| **Frequency of SCC in last 12 months** |  |  |  |  |
| ≤5 | 7 (78) | 77 (72) | 0.5 (2) | 0.7 |
| 6 – 10 | 2 (22) | 25 (23) |  |  |
| 11 - 15 | 0 (0) | 5 (5) |  |  |
| **Pain Severity** |  |  |  |  |
| Mild pain (1 – 3) | 1 (9) | 2 (2) | 2.2 (2) | 0.3 |
| Moderate pain (4 – 6) | 5 (45) | 56 (50) |  |  |
| Severe pain (7 – 10) | 5 (45) | 54 (48) |  |  |
| **Age of initial diagnosis** |  |  |  |  |
| Birth | 1 (10) | 9 (8) | 4.7 (4) | 0.3 |
| 0 - 5 years | 3 (30) | 52 (46) |  |  |
| 6 - 10 years | 3 (30) | 39 (35) |  |  |
| 11 - 15 years | 3 (30) | 10 (9) |  |  |
| 16+ years | 0 (0) | 2 (2) |  |  |
| **Level of Education** |  |  |  |  |
| Primary | 0 (0) | 11 (10) | 1.96 (2) | 0.4 |
| Secondary | 8 (73) | 83 (75) |  |  |
| University/Polytechnic/College of Education | 3 (27) | 17 (15) |  |  |
| **Occupation** |  |  |  |  |
| Student | 11 (100) | 108 (97) | 0.3 (1) | 0.6 |
| Others | 0 (0) | 3 (3) |  |  |
| **Religion** |  |  |  |  |
| Christianity | 8 (55) | 41 (38) | 1.3 (2) | 0.5 |
| Islam | 5 (45) | 67 (62) |  |  |
| Prefer not to say |  |  |  |  |

Less than five participants indicated they want to use deep-breathing exercises in the future. Hence, Chi-square analysis was not computed for future use of deep-breathing exercises.

# Distraction

| **Characteristics** | **Past Use of NPIs** | | | | **Future Use of NPIs** | | | |
| --- | --- | --- | --- | --- | --- | --- | --- | --- |
|  | **Users, n (%)** | **Non-users, n (%)** | **Pearson chi-square value (df)** | **P value** | **Users, n (%)** | **Non-users, n (%)** | **Pearson chi-square value (df)** | **P value** |
| **Age** |  |  |  |  |  |  |  |  |
| ≤15 years | 10 (56) | 65 (62) | 0.2 (1) | 0.6 | 5 (62) | 70 (61) | 0.004 (1) | 0.9 |
| >15 years | 8 (44) | 40 (38) |  |  | 3 (38) | 44 (39) |  |  |
| **Sex** |  |  |  |  |  |  |  |  |
| Female | 7 (39) | 53 (51) | 0.8 (1) | 0.4 | 3 (33) | 57 (50) | 0.9 (1) | 0.3 |
| Male | 11 (61) | 52 (49) |  |  | 9 (67) | 57 (50) |  |  |
| **Frequency of SCC in last 12 months** |  |  |  |  |  |  |  |  |
| ≤5 | 13 (77) | 71 (72) | 0.9 (2) | 0.6 | 9 (100) | 75 (70) | 3.7 (2) | 0.2 |
| 6 – 10 | 4 (23) | 23 (23) |  |  | 0 (0) | 27 (25) |  |  |
| 11 - 15 | 0 (0) | 5 (5) |  |  | 0 (0) | 5 (5) |  |  |
| **Pain Severity** |  |  |  |  |  |  |  |  |
| Mild pain (1 – 3) | 1 (6) | 2 (2) | 2.7 (2) | 0.2 | 1 (11) | 2 (2) | 5.2 (2) | 0.08 |
| Moderate pain (4 – 6) | 6 (33) | 55 (52) |  |  | 2 (22) | 59 (52) |  |  |
| Severe pain (7 – 10) | 11 (61) | 48 (46) |  |  | 6 (67) | 53 (46) |  |  |
| **Level of Education** |  |  |  |  |  |  |  |  |
| Primary | 0 (0) | 11 (11) | 2.8 (2) | 0.2 | 0 (0) | 11 (10) | 2.7 (2) | 0.3 |
| Secondary | 16 (89) | 75 (72) |  |  | 6 (67) | 85 (75) |  |  |
| University/Polytechnic/College of Education | 2 (11) | 18 (17) |  |  | 3 (33) | 17 (15) |  |  |
| **Age of initial diagnosis** |  |  |  |  |  |  |  |  |
| Birth | 1 (6) | 9 (8) | 4.5 (4) | 0.3 | 0 (0) | 10 (9) | 2.4 (4) | 0.6 |
| 0 - 5 years | 5 (29) | 50 (48) |  |  | 6 (67) | 49 (43) |  |  |
| 6 - 10 years | 7 (41) | 35 (33) |  |  | 2 (22) | 40 (35) |  |  |
| 11 - 15 years | 3 (8) | 10 (10) |  |  | 1 (11) | 12 (11) |  |  |
| 16+ years | 1 (6) | 1 (1) |  |  | 0 (0) | 2 (2) |  |  |
| **Occupation** |  |  |  |  |  |  |  |  |
| Student | 18 (100) | 101 (97) | 0.5 (1) | 0.5 | 8 (89) | 111 (98) | 3.0 (1) | 0.08 |
| Others | 0 (0) | 3 (3) |  |  | 1 (11) | 2 (2) |  |  |
| **Religion** |  |  |  |  |  |  |  |  |
| Christianity | 8 (44) | 39 (38) | 0.3 (2) | 0.8 | 5 (56) | 42 (37) | 1.2 (2) | 0.5 |
| Islam | 10(56) | 62 (61) |  |  | 4 (44) | 68 (61) |  |  |
| Prefer not to say | 0 (0) | 1 (1) |  |  | 0 (0) | 1 (1) |  |  |

# Herbal Products

| **Characteristics** | **Past Use of NPIs** | | | | **Future Use of NPIs** | | | |
| --- | --- | --- | --- | --- | --- | --- | --- | --- |
|  | **Users, n (%)** | **Non-users, n (%)** | **Pearson chi-square value (df)** | **P value** | **Users, n (%)** | **Non-users, n (%)** | **Pearson chi-square value (df)** | **P value** |
| **Age** |  |  |  |  |  |  |  |  |
| ≤15 years | 24 (71) | 51 (57) | 1.8 (1) | 0.2 | 27 (60) | 48 (62) | 0.7 (1) | 0.8 |
| >15 years | 10 (29) | 38 (43) |  |  | 18 (40) | 29 (38) |  |  |
| **Sex** |  |  |  |  |  |  |  |  |
| Female | 17 (50) | 43 (48) | 0.03 (1) | 0.9 | 21 (46) | 39 (51) | 0.3 (1) | 0.6 |
| Male | 17 (50) | 46 (52) |  |  | 25 (54) | 38 (49) |  |  |
| **Frequency of SCC in last 12 months** |  |  |  |  |  |  |  |  |
| ≤5 | 18 (58) | 66 (78) | 5.6 (2) | **0.05*** | 30 (67) | 54 (76) | 1.3 (2) | 0.5 |
| 6 – 10 | 12 (39) | 15 (17) |  |  | 13 (29) | 14 (20) |  |  |
| 11 - 15 | 1 (3) | 4 (5) |  |  | 2 (4) | 3 (4) |  |  |
| **Pain Severity** |  |  |  |  |  |  |  |  |
| Mild pain (1 – 3) | 0 (0) | 3 (3) | 1.2 (2) | 0.6 | 0 (0) | 3 (4) | 4.9 (2) | 0.09 |
| Moderate pain (4 – 6) | 17 (50) | 44 (49) |  |  | 28 (61) | 33 (43) |  |  |
| Severe pain (7 – 10) | 17 (50) | 42 (47) |  |  | 18 (39) | 41 (53) |  |  |
| **Level of Education** |  |  |  |  |  |  |  |  |
| Primary | 3 (9) | 8 (9) | 1.1 (2) | 0.9 | 6 (13) | 5 (7) | 3.5 (2) | 0.2 |
| Secondary | 24 (73) | 67 (75) |  |  | 30 (65) | 61 (80) |  |  |
| University/Polytechnic/College of Education | 6 (18) | 14 (16) |  |  | 10 (22) | 10 (13) |  |  |
| **Age of initial diagnosis** |  |  |  |  |  |  |  |  |
| Birth | 2 (6) | 8 (9) | 2.2 (4) | 0.7 | 6 (13) | 4 (5) | 3.9 (4) | 0.4 |
| 0 - 5 years | 16 (47) | 39 (44) |  |  | 22 (48) | 33 (43) |  |  |
| 6 - 10 years | 13 (38) | 29 (33) |  |  | 14 (30) | 28 (37) |  |  |
| 11 - 15 years | 3 (8) | 10 (11) |  |  | 3 (7) | 10 (13) |  |  |
| 16+ years | 0 (0) | 2 (2) |  |  | 1 (2) | 1 (1) |  |  |
| **Occupation** |  |  |  |  |  |  |  |  |
| Student | 33 (100) | 86 (97) | 1.1 (1) | 0.3 | 45 (98) | 74 (97) | 0.03 (1) | 0.9 |
| Others | 0 (0) | 3 (3) |  |  | 1 (2) | 2 (3) |  |  |
| **Religion** |  |  |  |  |  |  |  |  |
| Christianity | 9 (27) | 38 (44) | 3.2 (2) | 0.2 | 14 (31) | 33 (43) | 1.9 (2) | 0.3 |
| Islam | 24 (73) | 48 (55) |  |  | 30 (67) | 42 (55) |  |  |
| Prefer not to say | 0 (0) | 2 (2) |  |  | 1 (2) | 1 (1) |  |  |

# Local Heat

| **Characteristics** | **Past Use of NPIs** | | | | **Future Use of NPIs** | | | |
| --- | --- | --- | --- | --- | --- | --- | --- | --- |
|  | **Users, n (%)** | **Non-users, n (%)** | **Pearson chi-square value (df)** | **P value** | **Users, n (%)** | **Non-users, n (%)** | **Pearson chi-square value (df)** | **P value** |
| **Age** |  |  |  |  |  |  |  |  |
| ≤15 years | 13 (59) | 62 (61) | 0.04 (1) | 0.8 | 8 (53) | 67 (63) | 0.4 (1) | 0.5 |
| >15 years | 9 (41) | 39 (39) |  |  | 7 (47) | 40 (37) |  |  |
| **Sex** |  |  |  |  |  |  |  |  |
| Female | 11 (50) | 49 (49) | 0.02 (1) | 0.9 | 4 (25) | 56 (52) | 4.2 (1) | **0.04*** |
| Male | 11 (50) | 52 (52) |  |  | 12 (75) | 51 (48) |  |  |
| **Frequency of SCC in last 12 months** |  |  |  |  |  |  |  |  |
| ≤5 | 14 (70) | 70 (73) | 0.08 (2) | 0.9 | 10 (63) | 74 (74) | 0.9 (2) | 0.6 |
| 6 – 10 | 5 (25) | 22 (23) |  |  | 5 (31) | 22 (22) |  |  |
| 11 - 15 | 1 (5) | 4 (4) |  |  | 1 (6) | 4 (4) |  |  |
| **Pain Severity** |  |  |  |  |  |  |  |  |
| Mild pain (1 – 3) | 0 (0) | 3 (3) | 0.9 (2) | 0.6 | 0 (0) | 3 (3) | 1.8 (2) | 0.4 |
| Moderate pain (4 – 6) | 10 (46) | 51 (50) |  |  | 6 (38) | 55 (51) |  |  |
| Severe pain (7 – 10) | 12 (54) | 47 (47) |  |  | 10 (62) | 49 (46) |  |  |
| **Age of initial diagnosis** |  |  |  |  |  |  |  |  |
| Birth | 2 (10) | 8(8) | 13.5 (4) | **0.009*** | 0 (0) | 10 (9) | 3.6 (4) | 0.4 |
| 0 - 5 years | 5 (24) | 50 (50) |  |  | 10 (63) | 45 (42) |  |  |
| 6 - 10 years | 10 (48) | 32 (32) |  |  | 4 (25) | 38 (36) |  |  |
| 11 - 15 years | 2 (10) | 11 (11) |  |  | 2 (12) | 11 (10) |  |  |
| 16+ years | 2 (10) | 0 (0) |  |  | 0 (0) | 2 (2) |  |  |
| **Level of Education** |  |  |  |  |  |  |  |  |
| Primary | 1(5) | 10 (10) | 0.7 (2) | 0.7 | 0 (0) | 11 (10) | 1.8 (2) | 0.4 |
| Secondary | 17 (77) | 74 (74) |  |  | 13 (81) | 78 (74) |  |  |
| University/Polytechnic/College of Education | 4 (18) | 16 (16) |  |  | 3 (19) | 17 (16) |  |  |
| **Occupation** |  |  |  |  |  |  |  |  |
| Student | 21 (96) | 98 (97) | 0.5 (1) | 0.5 | 15 (94) | 104 (98) | 1.1 (1) | 0.2 |
| Others | 1 (4) | 2 (2) |  |  | 1 (6) | 2 (2) |  |  |
| **Religion** |  |  |  |  |  |  |  |  |
| Christianity | 15 (68) | 32 (33) | 9.5 (2) | **0.008*** | 8 (50) | 39 (37) | 3.7 (2) | 0.2 |
| Islam | 7 (32) | 65 (66) |  |  | 7 (44) | 65 (62) |  |  |
| Prefer not to say | 0 (0) | 1 (1) |  |  | 1 (6) | 1 (1) |  |  |

# Massage

| **Characteristics** | **Past Use of NPIs** | | | | **Future Use of NPIs** | | | |
| --- | --- | --- | --- | --- | --- | --- | --- | --- |
|  | **Users, n (%)** | **Non-users, n (%)** | **Pearson chi-square value (df)** | **P value** | **Users, n (%)** | **Non-users, n (%)** | **Pearson chi-square value (df)** | **P value** |
| **Age** |  |  |  |  |  |  |  |  |
| ≤15 years | 32 (65) | 43 (58) | 0.6 (1) | 0.4 | 24 (71) | 51 (58) | 1.6 (1) | 0.2 |
| >15 years | 17 (35) | 31 (42) |  |  | 10 (29) | 37 (42) |  |  |
| **Sex** |  |  |  |  |  |  |  |  |
| Female | 26 (53) | 34 (46) | 0.6 (1) | 0.4 | 17 (50) | 43 (48) | 0.03 (1) | 0.8 |
| Male | 23 (45) | 40 (54) |  |  | 17 (50) | 46 (52) |  |  |
| **Frequency of SCC in last 12 months** |  |  |  |  |  |  |  |  |
| ≤5 | 35 (78) | 49 (69) | 1.4 (2) | 0.5 | 27 (82) | 57 (69) | 3.1 (2) | 0.2 |
| 6 – 10 | 9 (20) | 18 (25) |  |  | 6 (18) | 21 (25) |  |  |
| 11 - 15 | 1 (2) | 4 (6) |  |  | 0 (0) | 5 (6) |  |  |
| **Pain Severity** |  |  |  |  |  |  |  |  |
| Mild pain (1 – 3) | 0 (0) | 3 (4) | 2.0 (2) | 0.4 | 1 (3) | 2 (2) | 2.5 (2) | 0.2 |
| Moderate pain (4 – 6) | 25 (51) | 36 (49) |  |  | 13 (38) | 48 (54) |  |  |
| Severe pain (7 – 10) | 24 (49) | 35 (47) |  |  | 20 (59) | 39 (44) |  |  |
| **Age of initial diagnosis** |  |  |  |  |  |  |  |  |
| Birth | 5 (10) | 5 (7) | 5.1 (4) | 0.3 | 2 (6) | 8 (9) | 2.7 (4) | 0.6 |
| 0 - 5 years | 18 (37) | 37 (51) |  |  | 18 (53) | 37 (42) |  |  |
| 6 - 10 years | 19 (39) | 23 (32) |  |  | 12 (35) | 30 (34) |  |  |
| 11 - 15 years | 5 (10) | 8 (11) |  |  | 2 (6) | 11 (13) |  |  |
| 16+ years | 2 (10) | 0 (0) |  |  | 0 (0) | 2 (2) |  |  |
| **Level of Education** |  |  |  |  |  |  |  |  |
| Primary | 3 (6) | 8 (11) | 1.6 (2) | 0.5 | 2 (6) | 9 (10) | 7.5 (2) | **0.02*** |
| Secondary | 36 (74) | 55 (75) |  |  | 31 (91) | 60 (68) |  |  |
| University/Polytechnic/College of Education | 10 (20) | 10 (14) |  |  | 1 (3) | 19 (22) |  |  |
| **Occupation** |  |  |  |  |  |  |  |  |
| Student | 47 (100) | 72 (97) | 0.9 (1) | 0.3 | 34 (100) | 85 (97) | 1.2 (1) | 0.3 |
| Others | 2 (0) | 1 (3) |  |  | 0 (0) | 3 (3) |  |  |
| **Religion** |  |  |  |  |  |  |  |  |
| Christianity | 25 (53) | 22 (30) | 6.8 (2) | **0.03*** | 14 (41) | 33 (38) | 0.9 (2) | 0.7 |
| Islam | 22 (47) | 50 (69) |  |  | 20 (59) | 52 (60) |  |  |
| Prefer not to say | 0 (0) | 1 (1) |  |  | 0 (0) | 2 (2) |  |  |

# Meditation

| **Characteristics** | **Past Use of NPIs** | | | | **Future Use of NPIs** | | | |
| --- | --- | --- | --- | --- | --- | --- | --- | --- |
|  | **Users, n (%)** | **Non-users, n (%)** | **Pearson chi-square value (df)** | **P value** | **Users, n (%)** | **Non-users, n (%)** | **Pearson chi-square value (df)** | **P value** |
| **Age** |  |  |  |  |  |  |  |  |
| ≤15 years | 6 (75) | 69 (60) | 0.7 (1) | 0.4 | 3 (50) | 72 (62) | 0.4 (1) | 0.6 |
| >15 years | 2 (25) | 46 (40) |  |  | 3 (50) | 44 (38 |  |  |
| **Sex** |  |  |  |  |  |  |  |  |
| Female | 5 (63) | 55 (48) | 0.6 (1) | 0.4 | 3 (50) | 57 (49) | 0.004 (1) | 0.9 |
| Male | 3 (37) | 60 (52) |  |  | 3 (50) | 60 (51) |  |  |
| **Frequency of SCC in last 12 months** |  |  |  |  |  |  |  |  |
| ≤5 | 7 (87) | 77 (71) | 1.1 (2) | 0.5 | 4 (67) | 80 (73) | 13.9 (2) | **<.001*** |
| 6 – 10 | 1 (13) | 26 (24) |  |  | 0 (0) | 27 (25) |  |  |
| 11 - 15 | 0 (0) | 5 (5) |  |  | 2 (33) | 3 (3) |  |  |
| **Pain Severity** |  |  |  |  |  |  |  |  |
| Mild pain (1 – 3) | 0 (0) | 3 (3) | 2.3 (2) | 0.3 | 0 (0) | 3 (3) | 3.2 (2) | 0.2 |
| Moderate pain (4 – 6) | 6 (75) | 55 (47) |  |  | 1 (17) | 60 (51) |  |  |
| Severe pain (7 – 10) | 2 (25) | 57 (50) |  |  | 5 (83) | 54 (46) |  |  |
| **Age of initial diagnosis** |  |  |  |  |  |  |  |  |
| Birth | 2 (25) | 8 (7) | 4.2 (4) | 0.3 | 0 (0) | 10 (9) | 0.9 (4) | 0.9 |
| 0 - 5 years | 4 (50) | 51 (45) |  |  | 3 (50) | 52 (45) |  |  |
| 6 - 10 years | 2 (25) | 40 (35) |  |  | 2 (33) | 40 (35) |  |  |
| 11 - 15 years | 0 (0) | 13 (11) |  |  | 1 (17) | 12 (10) |  |  |
| 16+ years | 2 (10) | 0 (0) |  |  | 0 (0) | 2 (2) |  |  |
| **Level of Education** |  |  |  |  |  |  |  |  |
| Primary | 0 (0) | 11 (10) | 2.9 (2) | 0.2 | 0 (0) | 11 (10) | 1.7 (2) | 0.4 |
| Secondary | 8 (100) | 83 (73) |  |  | 4 (67) | 87 (75) |  |  |
| University/Polytechnic/College of Education | 0 (0) | 20 (17) |  |  | 2 (33) | 18 (15) |  |  |
| **Occupation** |  |  |  |  |  |  |  |  |
| Student | 8 (100) | 111 (97) | 0.2 (1) | 0.6 | 6 (100) | 113 (97) | 0.2 (1) | 0.7 |
| Others | 0 (0) | 3 (3) |  |  | 0 (0) | 3 (3) |  |  |
| **Religion** |  |  |  |  |  |  |  |  |
| Christianity | 6 (75) | 41 (36) | 4.6 (2) | 0.09 | 6 (100) | 41 (36) | 9.9 (2) | **0.007*** |
| Islam | 2 (25) | 70 (63) |  |  | 0 (0) | 72 (63) |  |  |
| Prefer not to say | 0 (0) | 1 (1) |  |  | 0 (0) | 2 (2) |  |  |

# Music

| **Characteristics** | **Past Use of NPIs** | | | | **Future Use of NPIs** | | | |
| --- | --- | --- | --- | --- | --- | --- | --- | --- |
|  | **Users, n (%)** | **Non-users, n (%)** | **Pearson chi-square value (df)** | **P value** | **Users, n (%)** | **Non-users, n (%)** | **Pearson chi-square value (df)** | **P value** |
| **Age** |  |  |  |  |  |  |  |  |
| ≤15 years | 3 (60) | 72 (61) | 0.002 (1) | 0.9 | 3 (60) | 72 (62) | 0.005 (1) | 0.9 |
| >15 years | 2 (40) | 46 (39) |  |  | 2 (40) | 45 (38) |  |  |
| **Sex** |  |  |  |  |  |  |  |  |
| Female | 1 (20) | 59 (50) | 1.7 (1) | 0.2 | 3 (60) | 57 (48) | 0.3 (1) | 0.6 |
| Male | 4 (80) | 59 (50) |  |  | 2 (40) | 61 (52) |  |  |
| **Frequency of SCC in last 12 months** |  |  |  |  |  |  |  |  |
| ≤5 | 3 (70) | 81 (72) | 0.1 (2) | 0.9 | 4 (80) | 80 (72) | 0.3 (1) | 0.9 |
| 6 – 10 | 1 (25) | 26 (23) |  |  | 1 (20) | 26 (23) |  |  |
| 11 - 15 | 0 (0) | 5 (5) |  |  | 0 (0) | 5 (5) |  |  |
| **Pain Severity** |  |  |  |  |  |  |  |  |
| Mild pain (1 – 3) | 0 (0) | 3 (3) | 0.3 (2) | 0.8 | 0 (0) | 3 (3) | 0.3 (2) | 0.8 |
| Moderate pain (4 – 6) | 2 (40) | 59 (50) |  |  | 2 (40) | 59 (50) |  |  |
| Severe pain (7 – 10) | 3 (60) | 56 (47) |  |  | 3 (60) | 56 (47) |  |  |
| **Age of initial diagnosis** |  |  |  |  |  |  |  |  |
| Birth | 1 (20) | 9 (7) | 2.2 (2) | 0.7 | 0 (0) | 10 (9) | 1.5 (4) | 0.8 |
| 0 - 5 years | 1 (20) | 54 (46) |  |  | 3 (60) | 52 (44) |  |  |
| 6 - 10 years | 2 (40) | 40 (34) |  |  | 1 (20) | 41 (35) |  |  |
| 11 - 15 years | 1 (20) | 12 (10) |  |  | 1 (20) | 12 (10) |  |  |
| 16+ years | 0 (0) | 2 (10) |  |  | 0 (0) | 2 (2) |  |  |
| **Level of Education** |  |  |  |  |  |  |  |  |
| Primary | 0 (0) | 11 (9) | 2.4 (2) | 0.3 | 0 (0) | 11 (9) | 0.5 (2) | 0.8 |
| Secondary | 3 (73) | 88 (75) |  |  | 4 (80) | 87 (74) |  |  |
| University/Polytechnic/College of Education | 2 (27) | 18 (15) |  |  | 1 (20) | 19 (16) |  |  |
| **Occupation** |  |  |  |  |  |  |  |  |
| Student | 5 (100) | 114 (97) | 0.1 (1) | 0.7 | 5 (100) | 114 (97) | 0.1 (1) | 0.7 |
| Others | 0 (0) | 3 (3) |  |  | 0 (0) | 3 (3) |  |  |
| **Religion** |  |  |  |  |  |  |  |  |
| Christianity | 3 (60) | 44 (38) | 0.9 (2) | 0.6 | 4 (80) | 43 (37) | 3.7 (2) | 0.2 |
| Islam | 2 (40) | 70 (61) |  |  | 1 (20) | 71 (61) |  |  |
| Prefer not to say | 0 (0) | 1 (1) |  |  | 0 (0) | 2 (2) |  |  |

# Prayer

| **Characteristics** | **Past Use of NPIs** | | | | **Future Use of NPIs** | | | |
| --- | --- | --- | --- | --- | --- | --- | --- | --- |
|  | **Users, n (%)** | **Non-users, n (%)** | **Pearson chi-square value (df)** | **P value** | **Users, n (%)** | **Non-users, n (%)** | **Pearson chi-square value (df)** | **P value** |
| **Age** |  |  |  |  |  |  |  |  |
| ≤15 years | 15 (54) | 60 (63) | 0.8 (1) | 0.3 | 18 (62) | 57 (61) | 0.006 (1) | 0.9 |
| >15 years | 13 (46) | 35 (37) |  |  | 11 (38) | 36 (39) |  |  |
| **Sex** |  |  |  |  |  |  |  |  |
| Female | 10 (36) | 50 (53) | 2.5 (1) | 0.1 | 12 (40) | 48 (52) | 1.2 (1) | 0.3 |
| Male | 18 (64) | 45 (47) |  |  | 18 (60) | 45 (48) |  |  |
| **Frequency of SCC in last 12 months** |  |  |  |  |  |  |  |  |
| ≤5 | 18 (72) | 66 (73) | 0.02 (2) | 0.9 | 25 (86) | 59 (68) | 10.8 (2) | **0.004*** |
| 6 – 10 | 6 (24) | 21 (23) |  |  | 1 (3) | 26 (30) |  |  |
| 11 - 15 | 1 (4) | 4 (4) |  |  | 3 (10) | 2 (2) |  |  |
| **Pain Severity** |  |  |  |  |  |  |  |  |
| Mild pain (1 – 3) | 2 (7) | 1 (1) | 5.3 (2) | 0.06 | 2 (7) | 1 (1) | 3.9 (2) | 0.1 |
| Moderate pain (4 – 6) | 10 (36) | 51 (54) |  |  | 12 (40) | 49 (53) |  |  |
| Severe pain (7 – 10) | 16 (57) | 43 (45) |  |  | 16 (53) | 43 (46) |  |  |
| **Age of initial diagnosis** |  |  |  |  |  |  |  |  |
| Birth | 1 (4) | 9 (10) | 11.2 (4) | **0.02*** | 0 (0) | 10 (11) | 9.2 (4) | **0.05*** |
| 0 - 5 years | 8 (30) | 47 (50) |  |  | 9 (31) | 46 (50) |  |  |
| 6 - 10 years | 10 (37) | 32 (34) |  |  | 15 (52) | 27 (29) |  |  |
| 11 - 15 years | 7 (26) | 6 (6) |  |  | 4 (14) | 9 (10) |  |  |
| 16+ years | 1 (1) | 1 (4) |  |  | 1 (3) | 1 (1) |  |  |
| **Level of Education** |  |  |  |  |  |  |  |  |
| Primary | 0 (0) | 11 (12) | 3.9 (2) | 0.1 | 1 (3) | 10 (11) | 1.5 (2) | 0.5 |
| Secondary | 24 (86) | 67 (71) |  |  | 24 (80) | 67 (73) |  |  |
| University/Polytechnic/College of Education | 4 (14) | 16 (17) |  |  | 5 (17) | 15 (16) |  |  |
| **Occupation** |  |  |  |  |  |  |  |  |
| Student | 28 (100) | 91 (97) | 0.9 (1) | 0.3 | 28 (93) | 91 (99) | 2.9 (1) | 0.09 |
| Others | 0 (0) | 3 (3) |  |  | 2 (7) | 1 (1) |  |  |
| **Religion** |  |  |  |  |  |  |  |  |
| Christianity | 15 (56) | 32 (34) | 7.9 (2) | **0.02*** | 21 (70) | 26 (29) | 16.4 (2) | **<0.001*** |
| Islam | 11 (41) | 61 (66) |  |  | 9 (30) | 68 (69) |  |  |
| Prefer not to say | 1 (4) | 0 (0) |  |  | 2 (2) | 0 (0) |  |  |

*Significant

# Progressive muscle relaxation

| **Characteristics** | **Past Use of NPIs** | | | | **Future Use of NPIs** | | | |
| --- | --- | --- | --- | --- | --- | --- | --- | --- |
|  | **Users, n (%)** | **Non-users, n (%)** | **Pearson chi-square value (df)** | **P value** | **Users, n (%)** | **Non-users, n (%)** | **Pearson chi-square value (df)** | **P value** |
| **Age** |  |  |  |  |  |  |  |  |
| ≤15 years | 7 (58) | 68 (61) | 0.03 (1) | 0.8 | 6 (60) | 69 (62) | 0.01 (1) | 0.9 |
| >15 years | 5 (42) | 43 (39) |  |  | 4 (40) | 43 (38) |  |  |
| **Sex** |  |  |  |  |  |  |  |  |
| Male | 5 (42) | 55 (50) | 0.3 (1) | 0.6 | 4 (40) | 56 (50) | 0.3 (1) | 0.5 |
| Female | 7 (58) | 56 (51) |  |  | 6 (60) | 57 (50) |  |  |
| **Frequency of SCC in last 12 months** |  |  |  |  |  |  |  |  |
| ≤5 | 9 (75) | 75 (72) | 0.8 (2) | 0.7 | 5 (50) | 79 (75) | 2.9 (2) | 0.2 |
| 6 – 10 | 2 (17) | 25 (24) |  |  | 4 (40) | 23 (22) |  |  |
| 11 - 15 | 1 (8) | 4 (4) |  |  | 1 (10) | 4 (4) |  |  |
| **Pain Severity** |  |  |  |  |  |  |  |  |
| Mild pain (1 – 3) | 0 (0) | 3 (2) | 0.6 (2) | 0.7 | 0 (0) | 3 (3) | 0.8 (2) | 0.7 |
| Moderate pain (4 – 6) | 7 (58) | 54 (49) |  |  | 4 (40) | 57 (50) |  |  |
| Severe pain (7 – 10) | 5 (42) | 54 (49)) |  |  | 6 (60) | 53 (47) |  |  |
| **Level of Education** |  |  |  |  |  |  |  |  |
| Primary | 0 (0) | 11(10) | 1.8 (2) | 0.4 | 0 (0) | 11 (10) | 2.2 (2) | 0.3 |
| Secondary | 9 (75) | 82 (75) |  |  | 7 (70) | 84 (75) |  |  |
| University/Polytechnic/College of Education | 3 (25) | 17 (15) |  |  | 3 (30) | 17 (15) |  |  |
| **Age of initial diagnosis** |  |  |  |  |  |  |  |  |
| Birth | 1 (8) | 9 (8) | 5.5 (4) | 0.2 | 1 (10) | 9 (8) | 0.3 (4) | 0.9 |
| 0 - 5 years | 3 (25) | 52 (47) |  |  | 5 (50) | 50 (45) |  |  |
| 6 - 10 years | 5 (42) | 37 (34) |  |  | 3 (30) | 39 (35) |  |  |
| 11 - 15 years | 2 (17) | 11 (10) |  |  | 1 (10) | 12 (11) |  |  |
| 16+ years | 1 (1) | 1 (1) |  |  | 0 (0) | 2 (2) |  |  |
| **Occupation** |  |  |  |  |  |  |  |  |
| Student | 11 (92) | 119 (97) | 1.9 (1) | 0.1 | 10 (100) | 109 (97) | 0.3 (1) | 0.6 |
| Others | 1 (8) | 3 (3) |  |  | 0 (0) | 3 (3) |  |  |
| **Religion** |  |  |  |  |  |  |  |  |
| Christianity | 6 (55) | 41 (38) | 4.2 (2) | 0.1 | 43 (38) | 4 (44) | 0.3 (2) | 0.9 |
| Islam | 5 (45) | 67 (62) |  |  | 67 (60) | 5 (56) |  |  |
| Prefer not to say | 0 (0) | 1 (1) |  |  | 0 (0) | 2 (2) |  |  |

# Reading

| **Characteristics** | **Past Use of NPIs** | | | | **Future Use of NPIs** | | | |
| --- | --- | --- | --- | --- | --- | --- | --- | --- |
|  | **Users, n (%)** | **Non-users, n (%)** | **Pearson chi-square value (df)** | **P value** | **Users, n (%)** | **Non-users, n (%)** | **Pearson chi-square value (df)** | **P value** |
| **Age** |  |  |  |  |  |  |  |  |
| ≤15 years | 5 (63) | 70 (61) | 0.008 (1) | 0.9 | 6 (60) | 69 (62) | 0.01 (1) | 0.9 |
| >15 years | 3 (37) | 45 (39) |  |  | 4 (40) | 43 (38) |  |  |
| **Sex** |  |  |  |  |  |  |  |  |
| Female | 2 (25) | 58 (50) | 1.9 (1) | 0.2 | 6 (60) | 54 (48) | 0.5 (1) | 0.5 |
| Male | 6 (75) | 57 (57) |  |  | 4 (40) | 59 (52) |  |  |
| **Frequency of SCC in last 12 months** |  |  |  |  |  |  |  |  |
| ≤5 | 5 (71) | 79 (73) | 0.4 (2) | 0.8 | 7 (70) | 77 (73) | 7.1 (2) | **0.03*** |
| 6 – 10 | 2 (29) | 25 (23) |  |  | 1 (10) | 26 (25) |  |  |
| 11 - 15 | 0 (0) | 5 (5) |  |  | 2 (20) | 3 (3) |  |  |
| **Pain Severity** |  |  |  |  |  |  |  |  |
| Mild pain (1 – 3) | 1 (13) | 2 (2) | 3.8 (2) | 0.1 | 1 (10) | 2 (2) | 2.7 (2) | 0.3 |
| Moderate pain (4 – 6) | 3 (37) | 58 (50) |  |  | 5 (50) | 56 (50) |  |  |
| Severe pain (7 – 10) | 4 (50) | 55 (48) |  |  | 4 (40) | 55 (49) |  |  |
| **Level of Education** |  |  |  |  |  |  |  |  |
| Primary | 0 (0) | 11 (10) | 3.2 (2) | 0.1 | 1 (10) | 10 (9) | 0.3 (2) | 0.8 |
| Secondary | 5 (63) | 86 (75) |  |  | 8 (80) | 83 (74) |  |  |
| University/Polytechnic/College of Education | 3 (37) | 17 (15) |  |  | 1 (10) | 19 (17) |  |  |
| **Age of initial diagnosis** |  |  |  |  |  |  |  |  |
| Birth | 0 (0) | 10 (9) | 0.9 (4) | 0.9 | 0 (0) | 10 (9) | 7.5 (4) | 0.1 |
| 0 - 5 years | 4 (50) | 51 (57) |  |  | 4 (40) | 51 (50) |  |  |
| 6 - 10 years | 3 (38) | 39 (34) |  |  | 5 (50) | 37 (33) |  |  |
| 11 - 15 years | 1 (12) | 12 (11) |  |  | 0 (0) | 13 (12) |  |  |
| 16+ years | 0 (0) | 2 (2) |  |  | 1 (10) | 1 (1) |  |  |
| **Occupation** |  |  |  |  |  |  |  |  |
| Student | 8 (100) | 111 (97) | 0.2 (1) | 0.6 | 9 (90) | 110 (98) | 2.6 (1) | 0.1 |
| Others | 0 (0) | 3 (3) |  |  | 1 (10) | 2 (2) |  |  |
| **Religion** |  |  |  |  |  |  |  |  |
| Christianity | 4 (50) | 43 (38) | 0.4 (2) | 0.8 | 7 (70) | 40 (36) | 4.5 (3) | 0.2 |
| Islam | 4 (50) | 68 (61) |  |  | 3 (30) | 69 (62) |  |  |
| Prefer not to say | 0 (0) | 1 (1) |  |  | 0 (0) | 2 (2) |  |  |

# Relaxation

| **Characteristics** | **Past Use of NPIs** | | | | **Future Use of NPIs** | | | |
| --- | --- | --- | --- | --- | --- | --- | --- | --- |
|  | **Users, n (%)** | **Non-users, n (%)** | **Pearson chi-square value (df)** | **P value** | **Users, n (%)** | **Non-users, n (%)** | **Pearson chi-square value (df)** | **P value** |
| **Age** |  |  |  |  |  |  |  |  |
| ≤15 years | 7 (87) | 68 (59) | 2.5 (1) | 0.1 | 2 (40) | 73 (62) | 1.0 (1) | 0.3 |
| >15 years | 1 (13) | 47 (41) |  |  | 3 (60) | 44 (38) |  |  |
| **Sex** |  |  |  |  |  |  |  |  |
| Female | 3 (37) | 57 (50) | 0.4 (1) | 0.5 | 2 (33) | 58 (50) | 0.6 (1) | 0.4 |
| Male | 5 (63) | 58 (50) |  |  | 4 (67) | 59 (50) |  |  |
| **Frequency of SCC in last 12 months** |  |  |  |  |  |  |  |  |
| ≤5 | 6 (86) | 78 (72) | 0.8 (2) | 0.7 | 5 (83) | 79 (72) | 0.5 (2) | 0.8 |
| 6 – 10 | 1 (14) | 26 (24) |  |  | 1 (17) | 26 (24) |  |  |
| 11 - 15 | 0 (0) | 5 (5) |  |  | 0 (0) | 5 (5) |  |  |
| **Pain Severity** |  |  |  |  |  |  |  |  |
| Mild pain (1 – 3) | 0 (0) | 3 (2) | 0.7 (2) | 0.7 | 0 (0) | 3 (3) | 0.9 (2) | 0.6 |
| Moderate pain (4 – 6) | 5 (63) | 56 (49) |  |  | 2 (33) | 59 (50) |  |  |
| Severe pain (7 – 10) | 3 (37) | 56 (49) |  |  | 4 (67) | 55 (47) |  |  |
| **Level of Education** |  |  |  |  |  |  |  |  |
| Primary | 0 (0) | 11 (10) | 2.9 (2) | 0.2 | 0 (0) | 11 (10) | 1.7 (2) | 0.4 |
| Secondary | 8 (100) | 83 (72) |  |  | 4 (67) | 87 (75) |  |  |
| University/Polytechnic/College of Education | 0 (0) | 20 (18) |  |  | 2 (33) | 18 (15) |  |  |
| **Age of initial diagnosis** |  |  |  |  |  |  |  |  |
| Birth | 0 (0) | 10 (9) | 8.6 (4) | 0.07 | 0 (0) | 10 (9) | 3.9 (4) | 0.4 |
| 0 - 5 years | 5 (63) | 50 (44) |  |  | 5 (83) | 50 (43) |  |  |
| 6 - 10 years | 2 (25) | 40 (35) |  |  | 1 (17) | 41 (35) |  |  |
| 11 - 15 years | 0 (0) | 13 (11) |  |  | 0 (0) | 13 (11) |  |  |
| 16+ years | 1 (12) | 1 (1) |  |  | 0 (0) | 2 (2) |  |  |
| **Occupation** |  |  |  |  |  |  |  |  |
| Student | 8 (100) | 111 (97) | 0.2 (1) | 0.6 | 5 (83) | 114 (98) | 5.3 (1) | **0.02*** |
| Others | 0 (0) | 3 (3) |  |  | 1 (17) | 2 (2) |  |  |
| **Religion** |  |  |  |  |  |  |  |  |
| Christianity | 5 (71) | 44 (37) | 3.3 (2) | 0.1 | 5 (83) | 42 (37) | 5.3 (2) | 0.07 |
| Islam | 2 (29) | 70 (62) |  |  | 1 (17) | 71 (62) |  |  |
| Prefer not to say | 0 (0) | 1 (1) |  |  | 0 (0) | 2 (2) |  |  |

# Sleep/rest

| **Characteristics** | **Past Use of NPIs** | | | | **Future Use of NPIs** | | | |
| --- | --- | --- | --- | --- | --- | --- | --- | --- |
|  | **Users, n (%)** | **Non-users, n (%)** | **Pearson chi-square value (df)** | **P value** | **Users, n (%)** | **Non-users, n (%)** | **Pearson chi-square value (df)** | **P value** |
| **Age** |  |  |  |  |  |  |  |  |
| ≤15 years | 18 (90) | 57 (55) | 8.4 (1) | **0.004*** | 11 (55) | 64 (63) | 0.4 (1) | 0.5 |
| >15 years | 2 (10) | 46 (45) |  |  | 9 (45) | 38 (37) |  |  |
| **Sex** |  |  |  |  |  |  |  |  |
| Male | 8 (40) | 52 (60) | 0.7 (1) | 0.4 | 9 (43) | 51 (50) | 0.3 (1) | 0.6 |
| Female | 12 (60) | 51 (63) |  |  | 12 (57) | 51 (50) |  |  |
| **Frequency of SCC in last 12 months** |  |  |  |  |  |  |  |  |
| ≤5 | 14 (78) | 70 (71) | 1.0 (2) | 0.6 | 15 (75) | 69 (72) | 2.5 (2) | 0.3 |
| 6 – 10 | 4 (22) | 22 (24) |  |  | 3 (15) | 24 (25) |  |  |
| 11 - 15 | 0 (0) | 5 (5) |  |  | 2 (10) | 3 (3) |  |  |
| **Pain Severity** |  |  |  |  |  |  |  |  |
| Mild pain (1 – 3) | 2 (10) | 1 (1) | 5.7 (2) | 0.05* | 1 (5) | 2 (2) | 1.7 (2) | 0.4 |
| Moderate pain (4 – 6) | 9 (45) | 52 (50) |  |  | 8 (38) | 53 (52) |  |  |
| Severe pain (7 – 10) | 9 (45) | 52 (49) |  |  | 12 (57) | 47 (46) |  |  |
| **Level of Education** |  |  |  |  |  |  |  |  |
| Primary | 1 (5) | 10 (10) | 2.7 (2) | 0.2 | 3 (14) | 8 (8) | 4.1 (2) | 0.1 |
| Secondary | 17 (90) | 74 (72) |  |  | 12 (57) | 79 (78) |  |  |
| University/Polytechnic/College of Education | 1 (5) | 19 (18) |  |  | 6 (29) | 14 (14) |  |  |
| **Age of initial diagnosis** |  |  |  |  |  |  |  |  |
| Birth | 0 (0) | 10 (10) | 2.9 (4) | 0.6 | 1 (5) | 9 (9) | 5.1 (4) | 0.3 |
| 0 - 5 years | 11 (55) | 44 (43) |  |  | 13 (62) | 42 (42) |  |  |
| 6 - 10 years | 7 (35) | 35 (34) |  |  | 5 (24) | 37 (37) |  |  |
| 11 - 15 years | 2 (10) | 11 (11) |  |  | 1 (5) | 12 (12) |  |  |
| 16+ years | 0 (0) | 2 (2) |  |  | 1 (5) | 1 (1) |  |  |
| **Occupation** |  |  |  |  |  |  |  |  |
| Student | 19 (100) | 100 (97) | 0.6 (1) | 0.4 | 19 (91) | 100 (99) | 5.3 (1) | **0.02*** |
| Others | 0 (0) | 3 (3) |  |  | 2 (9) | 1 (1) |  |  |
| **Religion** |  |  |  |  |  |  |  |  |
| Christianity | 11 (58) | 36 (36) | 3.4 (2) | 0.2 | 12 (57) | 35 (35) | 3.8 (2) | 0.2 |
| Islam | 8 (42) | 64 (63) |  |  | 9 (43) | 63 (63) |  |  |
| Prefer not to say | 0 (0) | 1 (1) |  |  | 0 (0) | 2 (2) |  |  |

# Warm bath

| **Characteristics** | **Past Use of NPIs** | | | | **Future Use of NPIs** | | | |
| --- | --- | --- | --- | --- | --- | --- | --- | --- |
|  | **Users, n (%)** | **Non-users, n (%)** | **Pearson chi-square value (df)** | **P value** | **Users, n (%)** | **Non-users, n (%)** | **Pearson chi-square value (df)** | **P value** |
| **Age** |  |  |  |  |  |  |  |  |
| ≤15 years | 11 (61) | 64 (61) | 0 (1) | 0.9 | 9 (56) | 66 (62) | 0.2 (1) | 0.6 |
| >15 years | 7 (39) | 41 (39) |  |  | 7 (44) | 40 (38) |  |  |
| **Sex** |  |  |  |  |  |  |  |  |
| Male | 7 (39) | 53 (50) | 0.8 (1) | 0.4 | 6 (38) | 54 (51) | 0.9 (1) | 0.3 |
| Female | 11 (61) | 52 (50) |  |  | 10 (62) | 53 (49) |  |  |
| **Frequency of SCC in last 12 months** |  |  |  |  |  |  |  |  |
| ≤5 times | 11 (73) | 73 (72) | 0.8 (2) | 0.6 | 12 (75) | 72 (72) | 3.8 (2) | 0.1 |
| 6 – 10 | 4 (27) | 23 (23) |  |  | 2 (12) | 25 (25) |  |  |
| 11 - 15 | 0 (0) | 5 (5) |  |  | 2 (12) | 3 (3) |  |  |
| **Pain Severity** |  |  |  |  |  |  |  |  |
| Mild pain (1 – 3) | 1 (6) | 2 (2) | 1.3 (2) | 0.5 | 1 (6) | 2 (2) | 1.2 (2) | 0.5 |
| Moderate pain (4 – 6) | 10 (56) | 51 (48) |  |  | 8 (50) | 53 (50) |  |  |
| Severe pain (7 – 10) | 7 (38) | 52 (50) |  |  | 7 (44) | 52 (49) |  |  |
| **Level of Education** |  |  |  |  |  |  |  |  |
| Primary | 0 (0) | 11 (11) | 2.3 (2) | 0.3 | 1 (7) | 10 (9) | 1.4 (2) | 0.4 |
| Secondary | 14 (78) | 77 (74) |  |  | 13 (86) | 78 (73) |  |  |
| University/Polytechnic/College of Education | 4 (22) | 16 (15) |  |  | 1 (7) | 19 (18) |  |  |
| **Age of initial diagnosis** |  |  |  |  |  |  |  |  |
| Birth | 0 (0) | 10 (10) | 5.5 (4) | 0.2 | 0 (0) | 10 (9) | 4.7 (4) | 0.3 |
| 0 - 5 years | 7 (39) | 48 (46) |  |  | 7 (44) | 48 (45) |  |  |
| 6 - 10 years | 10 (56) | 32 (31) |  |  | 7 (44) | 35 (33) |  |  |
| 11 - 15 years | 1 (6) | 12 (11) |  |  | 1 (6) | 12 (11) |  |  |
| 16+ years | 0 (0) | 2 (2) |  |  | 1 (6) | 1 (1) |  |  |
| **Occupation** |  |  |  |  |  |  |  |  |
| Student | 18 (100) | 101 (97) | 0.5 (1) | 0.4 | 14 (93) | 105 (98) | 1.3 (1) | 0.3 |
| Others | 0 (0) | 3 (3) |  |  | 1 (7) | 2 (2) |  |  |
| **Religion** |  |  |  |  |  |  |  |  |
| Christianity | 7 (41) | 40 (39) | 0.2 (2) | 0.9 | 8 (50) | 39 (37) | 3.8 (2) | 0.2 |
| Islam | 10 (59) | 65 (66) |  |  | 7 (44) | 65 (62) |  |  |
| Prefer not to say | 0 (0) | 1 (1) |  |  | 1 (6.3) | 1 (1) |  |  |

# Other interventions

The following interventions had less than five adolescents using them hence chi-square analysis were not conducted:

1. Relaxation delivered through VR
2. Physical Exercises
3. Infrared radiation
4. Massage with Olive oil
5. Massage with Palm kernel oil
6. Massage with Robb
7. Massage with Shea butter
8. Tomato paste with coke
9. Forever Living Products
10. Watching football
11. Virtual reality
12. Yoga
13. Guided Imagery
14. Hypnosis
15. Play with pet
